# Synaptonemal Complex dimerization regulates chromosome alignment and crossover patterning in meiosis

**DOI:** 10.1371/journal.pgen.1009205

**Published:** 2021-03-17

**Authors:** Spencer G. Gordon, Lisa E. Kursel, Kewei Xu, Ofer Rog

**Affiliations:** School of Biological Sciences, University of Utah, Salt Lake City, Utah, United States of America; Stowers Institute for Medical Research, UNITED STATES

## Abstract

During sexual reproduction the parental homologous chromosomes find each other (pair) and align along their lengths by integrating local sequence homology with large-scale contiguity, thereby allowing for precise exchange of genetic information. The Synaptonemal Complex (SC) is a conserved zipper-like structure that assembles between the homologous chromosomes, bringing them together and regulating exchanges between them. However, the molecular mechanisms by which the SC carries out these functions remain poorly understood. Here we isolated and characterized two mutations in the dimerization interface in the middle of the SC zipper in *C*. *elegans*. The mutations perturb both chromosome alignment and the regulation of genetic exchanges. Underlying the chromosome-scale phenotypes are distinct alterations to the way SC subunits interact with one another. We propose a model whereby the SC brings homologous chromosomes together through two activities: obligate zipping that prevents assembly on unpaired chromosomes; and a tendency to extend pairing interactions along the entire length of the chromosomes.

## Author summary

Meiosis is a specialized cell division cycle during which maternal and paternal chromosomes are brought together, exchange information and separate into new cells that become gametes, such as sperm, egg or pollen. The Synaptonemal Complex (SC) is a zipper-like structure that assembles between the paternal and maternal chromosomes during meiosis, brings them together and regulates interactions between them. However, our understanding of structure/function relationships within the SC remains limited. Here we focused on one protein in the SC in nematodes—SYP-1—and isolated many random mutations in one of its domains. Two of these mutations had distinct effects on the way chromosomes were brought together and exchanged information. By characterizing these effects we came up with a model that links molecular-scale events in the middle of the SC zipper to chromosome-wide regulation of pairing and genetic exchanges. Future work defining the ultrastructure of the SC in the mutants will allow us to test the predictions of our model and define the mechanism by which the SC carries out its functions.

## Introduction

In meiotic cells, homologs are regulated precisely and synchronously as a pair via direct physical interaction, enabling them to exchange genetic information. For these interactions to occur, meiotic chromosomes undergo large scale conformational changes. First, each homolog becomes elongated and assembles onto a stiff proteinaceous structure called the axis. Then, homologs pair and align from end to end. Finally, homologs exchange genetic information through generation of crossovers. Crossovers also provide the physical linkages that hold the homologs together and allow them to segregate correctly into the gametes. Therefore, failure to bring the homologs together, and the subsequent absence of crossovers, result in aneuploid gametes and sterility.

Homolog alignment and regulation of crossovers rely on the assembly of a specialized interface called the Synaptonemal Complex (SC). (Throughout, we refer to the protein interface assembling between the axes, which is classically defined as the Central Region of the SC, simply as ‘the SC’). The SC loads at sites of localized interactions and processively zips up and aligns homologs along their entire length [1–3]. In addition to aligning homologs, the SC is required for both crossover formation, through recruitment of pro-crossover factors, and for regulating crossover number and distribution [4–6]. One manifestation of this regulation is wide spacing of crossovers through ‘crossover interference’. In *C*. *elegans*, interference ensures that, regardless of the number of DNA breaks, only one break per chromosome is repaired as a crossover [4].

**Fig 1:**
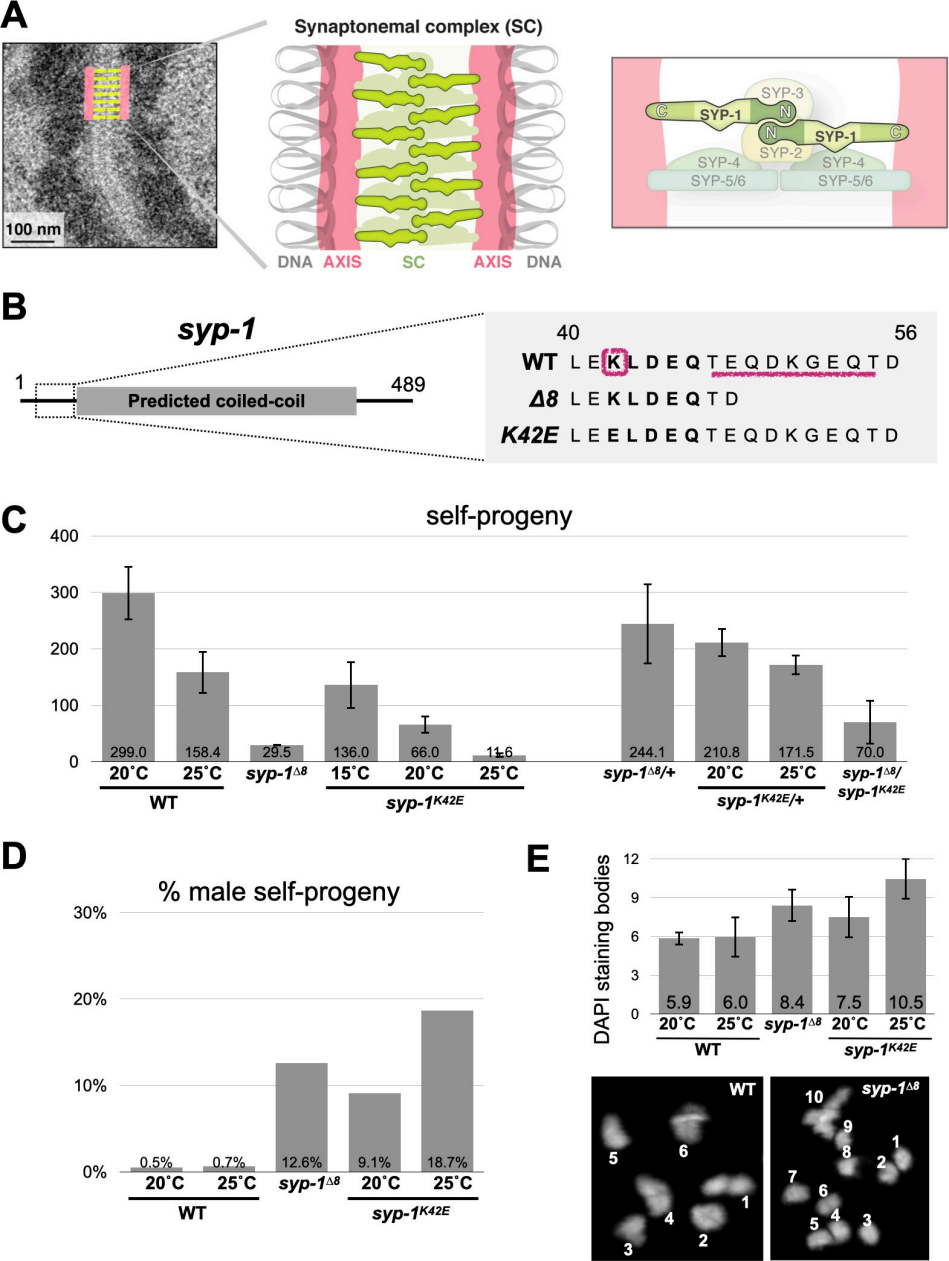
Isolation of separation-of-function mutations in the N-terminus of SYP-1. A) Model of the SC. Left, electron micrograph of *C*. *elegans* SC (adapted from [17]) with an overlaid interpretive diagram. The electron dense mass to the sides of the SC is chromatin. Middle, cartoon drawing of the SC. Parallel axes (salmon) organize the chromatin (gray) of each homolog into an elongated structure. The SC (green) holds the two homologs in alignment. Right, the stereotypic organization of SC components (from [11–13,15]). The right and left sides of the SC are mirror image of each other. The N- and C-termini of SYP-1 are labeled. B) *syp-1* gene model and mutations. The predicted coiled-coil in the middle is denoted by a gray bar. The conserved 5 amino acid stretch (KLEDQ at positions 42–46) is shown in bold. The residues mutated in *syp-1*^*K42E*^ and *syp-1*^*Δ8*^ are denoted with pink lines. See S1 Fig for more details. C) *syp-1* mutants show fertility defects. Self-progeny counts of wild-type and mutant hermaphrodites. Error bars indicate standard deviation. N>3 hermaphrodites for each condition. D) *syp-1* mutants show chromosome nondisjunction. Percent males among the self-progeny of wild-type and mutant hermaphrodites. Error bars indicate standard deviation. N>3 hermaphrodites for each condition. E) Chromosomes (“DAPI staining bodies”) at the diakinesis stage, immediately preceding the first meiotic division, are more numerous in *syp-1* mutants compared with wild type, indicating failure at forming crossovers on some chromosomes. Average DAPI staining body counts (reflecting the number of paired and unpaired chromosomes) from the indicated genotypes are shown. Error bars indicate standard deviation. N>14 nuclei for each condition. All pairwise comparisons are significant (Student’s t-test, p<0.05). Bottom, representative images of diakinesis nuclei with counted DAPI staining bodies from wild-type and *syp-1*^*Δ8*^ animals.

The SC ladder-like ultrastructure, as it appears in electron micrographs, is conserved across almost all eukaryotes with the two parallel axes separated by 100-150nm (Fig 1A; [3]). The SC is composed of a handful of subunits (six in *C*. *elegans* [7–12]) that occupy stereotypic locations relative to the axes (Fig 1A; [11–15]). However, we have limited understanding of how SC proteins interact with one another, or how their structure helps them to carry out functions such as aligning the homologs or regulating crossovers. Two main challenges have hampered progress. First, although SC ultrastructure and SC functions are conserved, the primary sequences of SC proteins diverge beyond recognition across eukaryotic lineages [3]. Second, attempts to purify SC components or express them *in vitro* have been mostly unsuccessful, and we therefore lack extensive structural and biochemical characterization of the SC [16]. Underlying the latter challenge might be the physical features of the SC: despite its appearance as an ordered, crystalline interface, the SC has recently been shown to have liquid-like properties and mobile subunits [17,18].

Null mutants of single SC components have not been very informative for elucidating structure-function relationships because they commonly abolish the SC altogether [7–12]. Genetic analyses in multiple model systems, however, have shown that the width of the SC is determined, at least in part, by the length of predicted coiled-coils in SC subunits referred to as transverse filament proteins [19–21], and that the C-termini of these proteins are required for axis associations [22,23]. In worms these transverse filament proteins include SYP-1 and SYP-5/6, which span, in a head-to-head orientation, the ~100 nm between the axes [7,11,12]. The contributions of other domains to specific functions or structural features are much less clear. For example, the ability of the SC to hold paired axes together was suggested to involve, based on *in vitro* analysis, dimerization and cooperative assembly of the N-terminus of the mammalian transverse filament protein SYCP1 [16], and based on genetic data, the N-terminus of budding yeast Zip1p [24,25]. However, while several genetic perturbations in *C*. *elegans* cause the SC to be associated with all chromosomes without holding paired axes together [10,26,27], the molecular details underlying pairing interactions have not been elucidated. Likewise, despite the identification of conditions that decrease crossover interference–mutations in the C-termini of *syp*-*4*, -*5* and -*6*, and partial depletion of SC components [4,11,13]–the molecular mechanisms that generate this chromosome-scale phenotype remain unknown.

To gain mechanistic insight into SC functions, we randomly mutagenized an N-terminal stretch of the SC component SYP-1, which localizes to the middle of the SC (Fig 1A; [5,13,15]). Detailed analyses of the separation-of-function mutations that we isolated showed that the N-terminus of SYP-1 is critical for the coupling of SC assembly and chromosome alignment, as well as for the regulation of crossovers.

## Results

### Generating SC hypomorphs by mutating the conserved N-terminus of SYP-1

Alignment of SYP-1 sequences from 10 *Caenorhabditis* species revealed that, despite the low overall sequence conservation, some regions are conserved. These include a phosphorylation site near the C-terminus [28,29] and sequences required for N-acetylation ([30] and S1A and S1B Fig). We focused on a conserved five amino acid stretch at the N-terminus of SYP-1 near the beginning of the predicted coiled-coil (Fig 1B). We generated mutations by randomly substituting single amino acids using CRISPR/Cas9, giving rise to 50 heterozygous mutants. We then attempted to homozygose these mutants, and eliminated those that were rendered sterile, assuming them to be *syp-1* null mutations [7]. We sequenced the remaining mutants and analyzed them for meiotic defects (Figs 1B and S1C). Interestingly, many non-conservative substitutions did not exhibit obvious meiotic phenotypes. However, two mutants stood out: a lysine to glutamic acid substitution at position 42 (*syp-1*^*K42E*^), and a serendipitous eight amino acid deletion immediately downstream of the conserved stretch we targeted for mutagenesis (*syp-1*^*Δ8*^; Fig 1B).

Both mutants exhibited hallmarks of meiotic defects. Self-progeny viability was dramatically reduced, and was accompanied by a high ratio of male progeny indicative of X chromosome missegregation (Fig 1C and 1D). The number of linked chromosomes prior to the meiotic divisions (‘DAPI staining bodies’) was larger than the six pairs observed in wild-type animals (Fig 1E). Notably, we found *syp-1*^*K42E*^ to be temperature sensitive. Progeny of *syp-1*^*K42E*^ animals exhibited progressively decreased viability and increased incidence of males with increasing temperature. At 25°C, a temperature in which meiosis in wild-type animals is mostly unaffected, *syp-1*^*K42E*^ animals had almost as few viable progeny as animals lacking an SC altogether [7].

### The N-terminus of SYP-1 is required for complete synapsis

**Fig 2:**
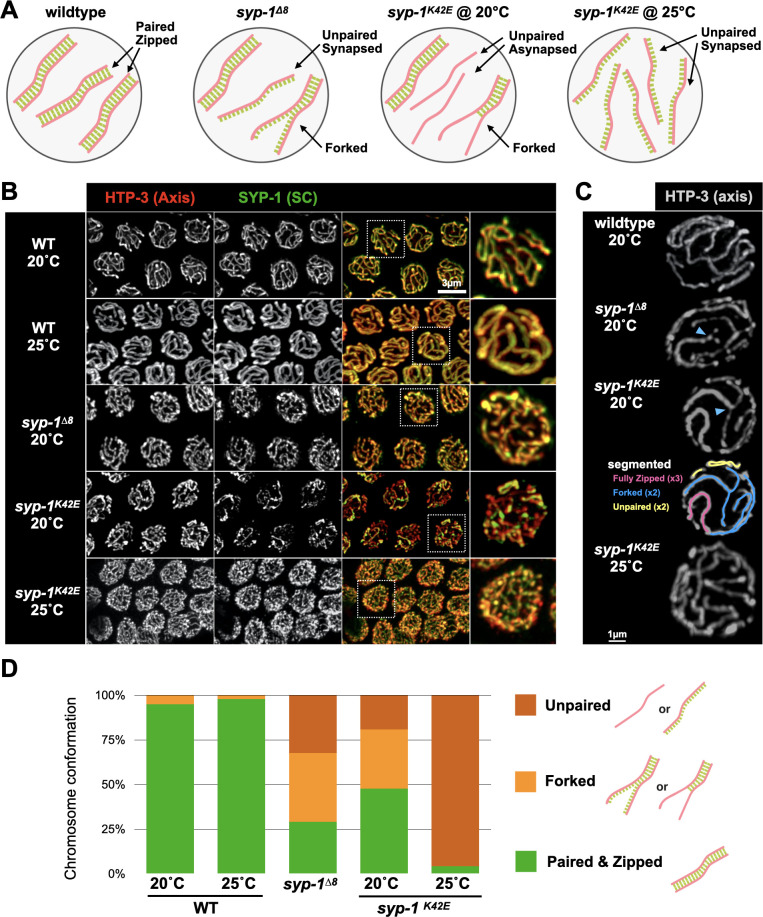
*syp-1* mutants display distinct defects in SC assembly. A) Model of chromosomal organization and SC association in *syp-1* mutants. For simplicity, only three homolog pairs are shown. Salmon, chromosome axis; green, SC; gray; nuclear envelope. B) Pachytene nuclei from wild-type worms and *syp-1* mutants showing synapsis defects. Nuclei are labeled with antibodies against the axis protein HTP-3 (red) and the SC (anti-SYP-1 antibodies; green). Higher magnification images are shown to the right. Notably, the SC fails to fully associate with all chromosomes in *syp-1*^*K42E*^ at 20°C, as revealed by axis signal not associated with SC. Note the numerous SC threads in *syp-1*^*Δ8*^ and *syp-1*^*K42E*^ (25°C), caused by the unpaired chromosomes still associated with the SC. Scale bar = 3μm. C) Partial projections of detergent-extracted pachytene nuclei from wild type and *syp-1* mutants labeled with anti-HTP-3 (gray). The enhanced resolution allows tracing of the different synapsis outcomes, shown for *syp-1*^*K42E*^ (20°C; skeletonized segments): fully zipped (pink), forked (blue), and unpaired and asynapsed (yellow). Forked chromosomes are indicated with a blue arrowhead. Scale bar = 1μm. D) Summary of the different chromosomal conformation in pachytene nuclei from wild-type and *syp-1* mutant worms. Only chromosomes that were clearly laid out on the xy-plane of partially projected gonads were scored. The density of unpaired chromosomes in *syp-1*^*K42E*^ (25°C) animals made them difficult to count; therefore, only zipped chromosomes were counted and all other chromosomes were assumed to be unpaired. N>95 chromosomes from at least 3 different gonads in each condition. All pairwise comparisons to wild type are significant (Pearson’s chi-squared test, where ‘unpaired’ and ‘forked’ configurations were grouped together; p<0.0001).

The term ‘synapsis’ has historically been used to indicate both association of the SC with the axis *and* alignment of two axes concomitant with SC assembly. This is indeed the case in wild-type animals, where the SC assembles only between the paired axes, aligning the homologs from end to end. The phenotypes of our mutants (detailed below and summarized in Fig 2A) show that these two SC activities can be separated. We therefore use the term ‘synapsis’ to describe the association of SC and axes, ‘zipping’ for SC-mediated alignment of the two axes, and ‘pairing’ to describe the association between the homologs. In *syp-1*^*Δ8*^ animals zipping is partially defective and synapsis occurs with unpaired axes. Zipping is also defective in *syp-1*^*K42E*^ animals. At 20°C the SC fails to fully zip the homologs but synapsis is still exclusive to paired axes. However, at 25°C zipping is nearly abolished, and this is accompanied by widespread synapsis with unpaired axes.

To analyze synapsis in our mutants, we visualized the SC and the axes in meiotic prophase nuclei using antibodies against SYP-1 and the axis protein HTP-3. In wild-type animals the SC assembles on all chromosomes, thereby colocalizing the SYP-1 and HTP-3 signals (Fig 2B). In *syp-1*^*K42E*^ (20°C) animals, some chromosomes were not synapsed, as evidenced by HTP-3 staining unaccompanied by SYP-1 staining (Fig 2B). Higher resolution analysis indicated that in addition to chromosomes that are completely asynapsed or completely zipped, some chromosomes were forked–zipped along part of their length with their ends splayed and asynapsed (Fig 2C and 2D). Given the severe meiotic defects, we were initially surprised to observe complete colocalization of SC and axes in *syp-1*^*K42E*^ (25°C) and in *syp-1*^*Δ8*^ animals (Fig 2B). Chromosome morphology, however, was distinct from wild-type animals. Chromosomes in *syp-1*^*K42E*^ (25°C) animals were almost all unzipped, with all unpaired axes associated with the SC (Fig 2C and 2D). This phenotype is reminiscent of mutants in the regulatory proteins HAL-2/3 [26,27], as well as the small C-terminal truncation in *syp-3(me42)* [10]. Chromosome axes in *syp-1*^*Δ8*^ were also all associated with the SC, but closer inspection revealed a combination of unzipped, forked, and fully zipped chromosomes, similar to the conformations observed in *syp-1*^*K42E*^ (20°C; Fig 2C and 2D). Confirming the presence of unzipped but SC-associated chromosomes, the SC localized to the X chromosomes in *syp-1*^*Δ8*^ animals where X chromosome pairing was prevented by deletion of *him-8* ([31]; S2A Fig). Notably, we did not observe non-continuous SC stretches or bubbles in any mutant scenario, consistent with cooperative assembly of the SC [32].

**Fig 3:**
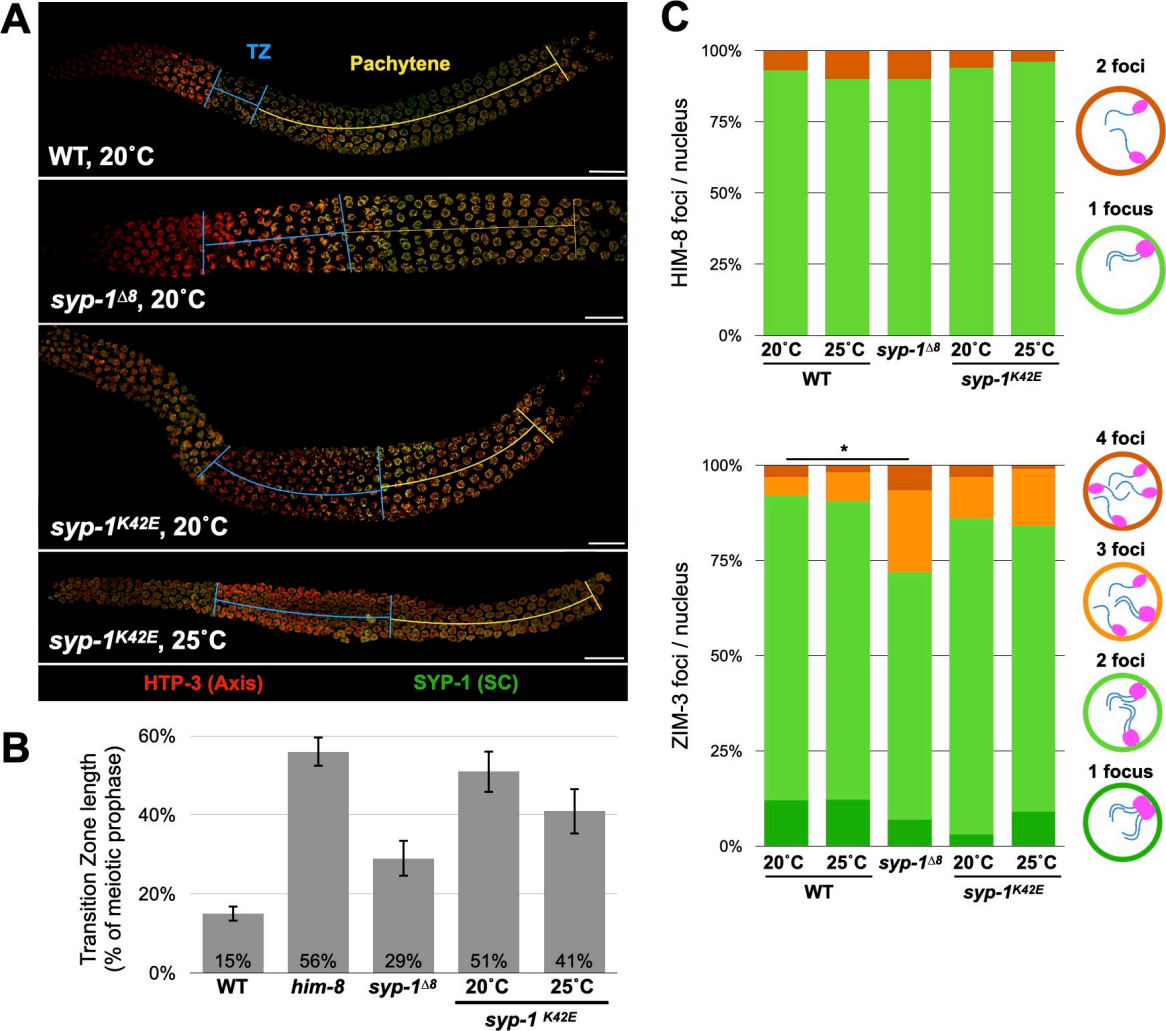
*syp-1* mutants display distinct defects in meiotic progression. A) Immunofluorescence images of axis (anti-HTP-3, red) and SC (anti-SYP-1, green) in the indicated genotypes showing elongated transition zone in *syp-1* mutants. Nuclei progress in the gonad from left to right as they progress in meiosis. Blue lines correspond to the transition zone based on chromatin morphology and yellow lines designate pachytene. Scale bars = 15μm. B) Quantitation of the transition zone length, calculated as percentages of the gonad relative to the entirety of meiotic prophase. *him-8* animals, where the X chromosome fails to pair and synapse, are shown as a control for highly extended transition zone [31]. Error bars indicate standard deviation. N>4 gonads for each condition. All pairwise comparisons to wild type are significant (Student’s t-test, p<0.05). C) Pairing at Pairing Centers is not dramatically affected in *syp-1* mutants. Top, X chromosome pairing shown by localization of the Pairing Center protein HIM-8 in animals of the indicated genotypes. Paired and unpaired X chromosomes are shown as one or two HIM-8 foci, respectively. Models are shown to the right. N>130 nuclei in each condition. Pairwise comparisons to wild type are not significant (Pearson’s chi-squared test). Bottom, pairing of chromosomes I and IV shown by localization of the Pairing Center protein ZIM-3 in animals of the indicated genotypes. Correctly paired chromosomes I and IV would result in one or two foci (green), whereas three or four foci indicate incorrect pairing (orange). Models are shown to the right. N>90 nuclei from at least three gonads in each condition. Significant pairwise comparisons to wild type are indicated (Pearson’s chi-squared test, comparing ‘correct’ versus ‘incorrect’ configurations; p<0.0005).

Synapsis defects and exposed chromosome axes have been associated with changes in meiotic progression [33]. Consistent with this, we observed a dramatically extended duration of the leptotene-zygotene stage of meiosis (“transition zone”; Fig 3A and 3B) in *syp-1*^*K42E*^ (20°C) animals. *syp-1*^*Δ8*^ and *syp-1*^*K42E*^ (25°C) animals had a more mildly extended transition zone despite seemingly complete colocalization of axis and SC staining, which might reflect slow association of the SC with the axis or an altered SC ultrastructure that is not evident at the resolution of light microscopy. We also quantified the relative abundance of an axis and an SC component in wild-type and mutant animals and found only minor effects on SC abundance (S2B Fig), arguing that reduced SC protein levels cannot account for the phenotypes we observe.

We confirmed that the zipped chromosomes are indeed homologs. In *C*. *elegans*, homolog pairing is promoted by sites on each chromosome called Pairing Centers [31,34,35]. We found that Pairing Centers on chromosomes I, IV and X are mostly correctly paired in *syp-1*^*K42E*^ and *syp-1*^*Δ8*^ animals (Fig 3C), suggesting that homolog pairing is not dramatically impacted. Furthermore, since zipping by the SC is an essential precondition for crossover formation in worms [7], the presence of some linked chromosomes prior to the meiotic divisions (Fig 1E) suggests that the SC was assembling between homologs, and that heterologous synapsis is rare.

Our analysis demonstrated that despite their proximity on the N-terminus of SYP-1, our mutants cause distinct chromosomal phenotypes. Underlying these phenotypes are violations of two principles that govern SC assembly: loading only between paired axes, and spreading along the entire length of the chromosome. Association with only paired axes is disrupted in *syp-1*^*Δ8*^ and *syp-1*^*K42E*^ (25°C) animals; full zipping to align chromosomes end-to-end is disrupted in *syp-1*^*Δ8*^ and *syp-1*^*K42E*^ (20°C) animals.

### The N-terminus of SYP-1 is crucial for intra-SC interactions

**Fig 4:**
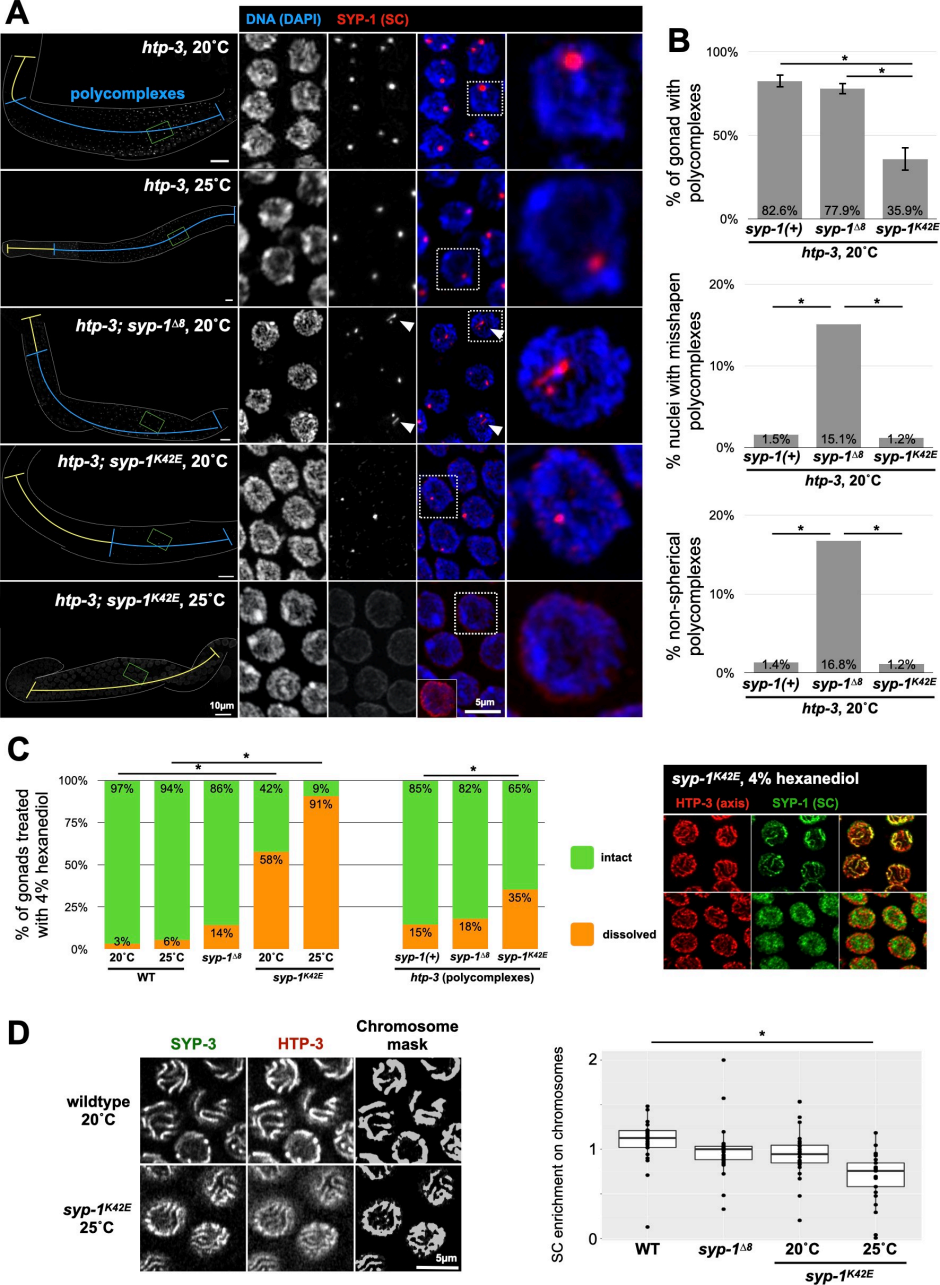
The N-terminus of SYP-1 plays a role in intra-SC interactions. A) Polycomplex morphology and dynamics are altered in *htp-3 syp-1*^*K42E*^ and *htp-3 syp-1*^*Δ8*^ animals. Immunofluorescence images of gonads stained with anti-SYP-1 antibodies. The region of the gonad containing nuclei with polycomplexes is marked with a blue line, whereas the yellow line designates meiotic nuclei without polycomplexes. Scale bars = 10μm. Middle, pachytene nuclei, with a magnified nucleus shown to the right. SYP-1 polycomplexes are red and DAPI (DNA) is shown in blue. Misshapen polycomplexes (arrowheads) occur in *htp-3 syp-1*^*Δ8*^ animals. *htp-3 syp-1*^*K42E*^ (25°C) harbor no polycomplexes; bottom left, over-exposed nucleus showing only diffuse SC staining. Scale bar = 5μm. B) Quantitation of polycomplexes in *syp-1* mutants. Top, the portion of the gonad harboring polycomplexes. Error bars indicate standard deviations. N>4 gonads in each condition. Significant pairwise comparisons are indicated (Student’s t-test, p<0.0005). Middle, percentage of nuclei containing misshapen polycomplexes, scored manually. N>160 nuclei from three different gonads for each genotype. Significant pairwise comparisons are indicated (Pearson’s chi-squared test, p<0.0005). Bottom, non-spherical polycomplexes (ratio between the short and long axis of less than 0.5, where 1 represent a perfect sphere). N>80 polycomplexes from at least three gonads for each genotype. Significant pairwise comparisons are indicated (Pearson’s chi-squared test, p<0.0005). See S3A Fig for additional data. C) Assembled SC and polycomplexes in *syp-1*^*K42E*^ animals are hypersensitive to dissolution by 4% 1,6-hexanediol. Left, percentage of gonads from the indicated genotypes where the SC was dissolved with 4% 1,6-hexanediol. Middle, percentage of gonads from the indicated genotypes where polycomplexes were dissolved with 4% 1,6-hexanediol. N>30 gonads for each condition. Significant pairwise comparisons to wild type or *syp-1(+)* are indicated (Pearson’s chi-squared test, p<0.05). Right, immunofluorescence images of representative nuclei with intact SC (chromosome associated, top) and dissolved SC (diffuse nucleoplasmic, bottom) in pachytene nuclei from *syp-1*^*K42E*^ (20°C) animals. D) *syp-1* mutants affect the enrichment of the SC on chromosomes versus nucleoplasm. Left, representative nuclei from wild-type and *syp-1*^*K42E*^ (25°C) animals expressing GFP-SYP-3 and HTP-3-wrmScarlet. A chromosome mask was calculated based on HTP-3-wrmScarlet (see Methods for details). Right, the enrichment ratio was calculated by dividing the sum GFP-SYP-3 intensity inside the chromosome mask by the sum intensity outside the mask, and normalizing by the enrichment value for HTP-3-wrmScarlet. The bars indicate the median enrichment, and the boxes indicate the middle 50%. N>30 nuclei, from at least three animals, for each condition. Significant comparisons to wild type are shown (p<0.0001; Student’s t-test).

We next wanted to understand how alterations in the N-terminus of SYP-1 bring about such dramatic chromosome-scale effects. Since the SC has proved challenging to reconstitute *in vitro*, we used several orthogonal approaches to measure the biophysical properties of the SC in our mutants. In many organisms, SC material can form organized structures called polycomplexes that appear as stacked SC [3,17,36]. In worms, polycomplexes arise when SC material cannot load onto chromosomes due to elimination of the axis protein HTP-3 [37]. In *htp-3* animals there is generally one spherical polycomplex per nucleus throughout meiotic prophase, until late pachytene when the number of polycomplexes per nucleus increases [17]. *syp-1* mutations alter this dynamic. Polycomplexes form later in *htp-3 syp-1*^*K42E*^ (25°C) animals, and at 25°C most worms do not form polycomplexes. Instead, *htp-3 syp-1*^*K42E*^ (20°C) animals exhibit diffuse SC material in the nucleoplasm, while a minority forms a few polycomplexes in late pachytene (Figs 4A and 4B and S3A). In contrast, *htp-3 syp-1*^*Δ8*^ polycomplexes appear with similar dynamics to those in *htp-3* animals, but are more numerous and are non-spherical, resembling in their appearance structures that form when another axis component, HIM-3, is removed [38].

We also tested the integrity of the SC, both assembled between chromosomes and as polycomplexes, using 1,6-hexanediol, an aliphatic alcohol that disrupts hydrophobic interactions and dissolves the SC at high concentrations [17]. Low concentrations of 1,6-hexanediol can be used to probe the strength of intra-SC interactions. In wild-type nuclei, the SC rarely dissolves when treated with 4% 1,6-hexanediol (1/31 gonads exhibited fully dissolved SC; Fig 4C). The SC in *syp-1*^*K42E*^ (20°C) animals dissolve more easily with 4% 1,6-hexanediol (41/71 gonads), an effect that is exacerbated at 25°C (29/32 gonads). The SC in *syp-1*^*Δ8*^ animals dissolve at a frequency much closer to wild type (10/70 gonads). Polycomplex dissolution correlated with that of assembled SC, and was significantly higher for *syp-1*^*K42E*^ (20°C) compared with wild-type polycomplexes (Fig 4C).

These analyses of SC and polycomplex integrity suggest that the SC in our mutants, especially in *syp-1*^*K42E*^ (25°C) animals, has weakened intra-SC interactions compared to wild type. We hypothesized that weaker affinity between SC subunits will result in less efficient recruitment of the SC onto chromosomes. That was indeed the case: the average enrichment of GFP-SYP-3 on chromosomes in pachytene nuclei relative to the nucleoplasm was ~2-fold lower in *syp-1*^*K42E*^ (25°C) compared with wild-type animals, with *syp-1*^*K42E*^ (20°C) and *syp-1*^*Δ8*^ animals exhibiting similar enrichment to wild type (Fig 4D).

Taken together, our analysis indicates that while the chromosomal phenotypes of our N-terminal *syp-1* mutations are related, they stem from different effects on the biophysical properties of the SC. *syp-1*^*K42E*^ weakens intra-SC interactions as reflected by poor assembly into polycomplexes (when loading onto axes is abolished), increased 1,6-hexanediol sensitivity, and inefficient recruitment onto chromosomes, all of which are exacerbated by increasing temperature. In contrast, *syp-1*^*Δ8*^ alters the SC in a way that involves only a minor weakening of SC integrity.

### The N-terminus of SYP-1 plays a role in crossover regulation

The SC regulates crossovers not only by aligning homologs and bringing homologous sequences in close proximity, but also in two additional ways: recruiting pro-crossover factors and regulating crossover distribution [4–6,17,24,25,39]. Both our *syp-1* mutants remain competent for recruiting pro-crossover factors: progeny viability and the number of DAPI staining bodies (Fig 1E) suggest that the SC in *syp-1*^*K42E*^ and *syp-1*^*Δ8*^ animals have not entirely lost the capacity to promote crossover formation. Furthermore, the stereotypic localization of the pro-crossover factor ZHP-3 to polycomplexes–indicative of crossover regulation by the SC independent of the axes or of repair intermediates–was unaffected ([17]; S3B Fig).

**Fig 5:**
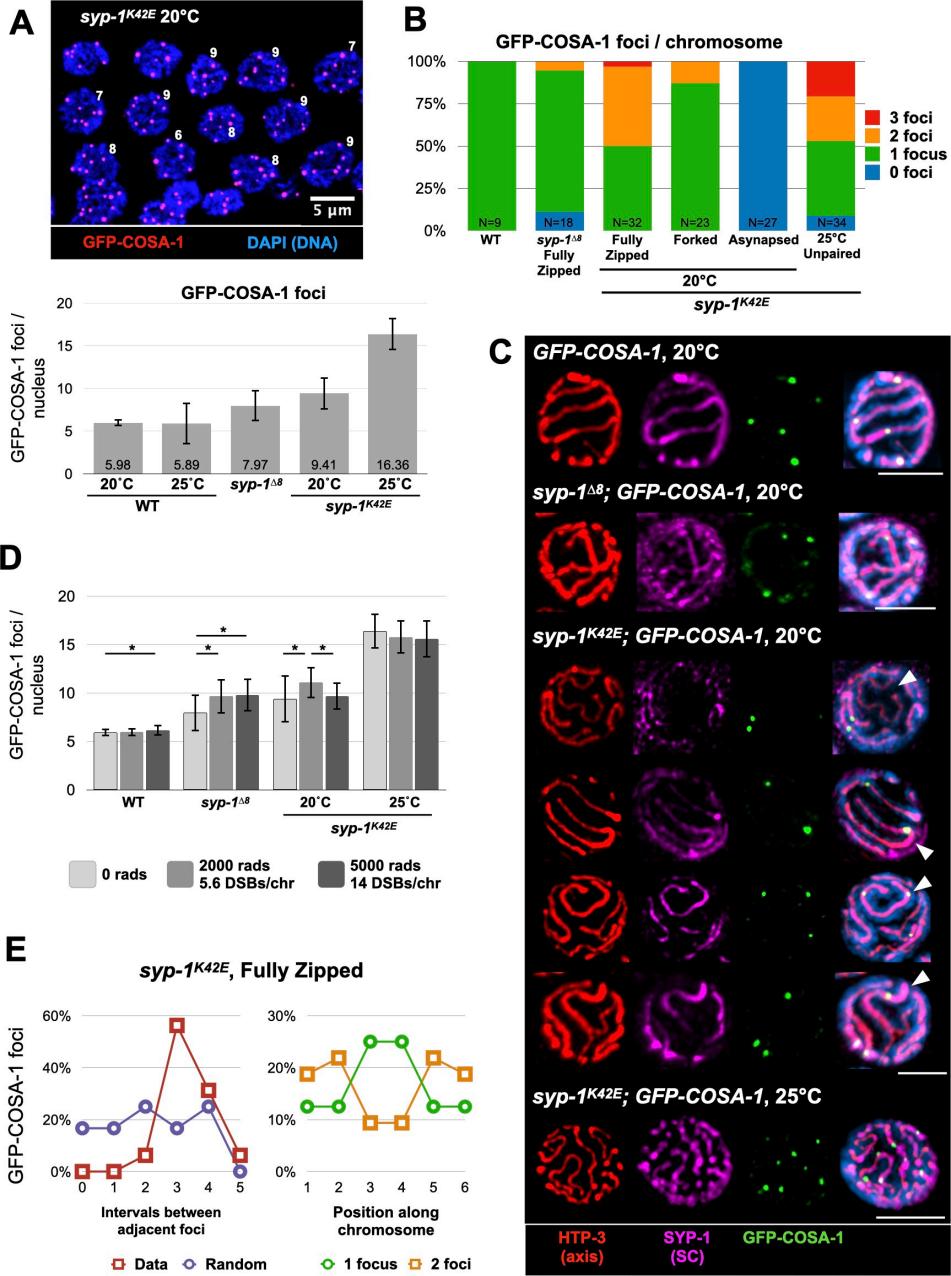
The N-terminus of SYP-1 regulates the number and distribution of crossover markers. A) GFP-COSA-1 foci are more numerous in *syp-1* mutants. Top, immunofluorescence images of late pachytene *syp-1*^*K42E*^ nuclei showing the number of GFP-COSA-1 foci per nucleus (red). Scale bar = 5μm. Bottom, the average number of GFP-COSA-1 foci per nucleus in the indicated genotypes. Error bars indicate standard deviation. N>30 nuclei from at least three gonads for each condition. All pairwise comparisons to wild type are significant (Student’s t-test, p<0.005). B) The number of GFP-COSA-1 foci per chromosome traced in 3D in the indicated genotypes. Chromosomes in *syp-1*^*K42E*^ animals are shown by the type of chromosome morphology (fully zipped, forked and asynapsed). N values are shown at the bottom of each bar. C) Examples of GFP-COSA-1 localization relative to the SC. Partial projections of immunofluorescence images of detergent-extracted pachytene nuclei form animals of the indicated genotypes. Nuclei were stained with antibodies against HTP-3 (red), SYP-1 (magenta) and GFP (green; GFP-COSA-1), and with DAPI (DNA; blue). Scale bars = 3μm. Notably, GFP-COSA-1 foci are associated with the SC in all scenarios. *syp-1*^*K42E*^*; GFP-COSA-1* (20°C): Top row, unpaired chromosome that is not associated with the SC (arrowhead) also does not recruit GFP-COSA-1. 2^nd^ row, fully zipped chromosome with a single GFP-COSA-1 focus (arrowhead). 3^rd^ row, a fully zipped chromosome with two foci (arrowhead). Bottom row, a forked chromosome (arrowhead) with a single GFP-COSA-1 focus in the zipped region. Throughout our images we found that GFP-COSA-1 foci were not enriched near forking junctions (only 1/25 junctions was associated with a focus, and only 4 of 25 foci (16%) were <1μm from a junction), arguing against the idea that crossovers locally stabilize the SC. *syp-1*^*K42E*^*; GFP-COSA-1* (25°C): Note multiple GFP-COSA-1 foci along single chromosomes. D) The number of GFP-COSA-1 foci is not limited by the number of double-strand breaks. GFP-COSA-1 foci in animals of the indicated genotypes after being exposed to varying amounts of X-ray irradiation (2000 and 5000 Rads), creating 5.6 and 14 extra breaks per chromosome, respectively. See S4 Fig for analysis of X-ray induced breaks. Error bars indicate standard deviation. N>20 nuclei from at least three gonads for all conditions. Significant intra-genotype comparisons are shown (Student’s t-test, p<0.05). E) Intact but reduced interference between crossover precursors. GFP-COSA-1 foci were identified along completely zipped chromosomes in *syp-1*^*K42E*^ (20°C) animals, and their positions were placed into six equal bins. Left, in chromosomes that harbor two foci, the foci are more distant from each other (maroon) than would be expected by chance (‘Random’, purple). Right, histograms of the position along the chromosome, normalized around the center of the chromosome. When 2 foci are present (orange), they tend to be at the opposite ends of the chromosomes. N = 15 chromosomes from at least three gonads.

While recruitment of pro-crossover factors remains intact, we wanted to assess whether the number and distribution of crossovers is affected in *syp-1* mutants. We counted GFP-COSA-1 foci, which are cytological markers of crossovers [40], and traced them along each chromosome. GFP-COSA-1 foci were more numerous in *syp-1*^*K42E*^ and *syp-1*^*Δ8*^ worms compared to the six foci observed in wild-type animals, one on each of the six homologs (Fig 5A–5C). This was particularly striking in the case of *syp-1*^*K42E*^ (25°C) animals, where despite lack of homolog zipping, and hence of actual crossovers, we observed an average of 16.4 GFP-COSA-1 foci per nucleus (Fig 5A–5C). Based on observations of differentially labeled sister-chromatids, at least some of these foci likely mark inter-sister exchanges [41]. Tracing of GFP-COSA-1 foci in *syp-1*^*Δ8*^ animals indicated that the increased overall foci number is not due to association of multiple foci per chromosome–like in wild-type animals, every chromosome harbors only a single focus–but likely due to association of foci with the SC on both zipped and unzipped chromosomes (Fig 5A–5C). In *syp-1*^*K42E*^ animals, at both 20°C and 25°C, many chromosomes harbored more than one GFP-COSA-1 focus, contributing to the large number of overall foci (Fig 5A–5C). To exclude the possibility that a limited number, or altered distribution, of meiotic double-strand breaks is shaping the crossover landscape in our mutants, we introduced as many as 14 additional breaks per chromosome by X-ray irradiation (S4 Fig). We observed only minor increases in the number of GFP-COSA-1 foci (Fig 5D), suggesting that perturbed crossover distribution in our mutants indeed reflects altered regulation by the SC.

**Fig 6:**
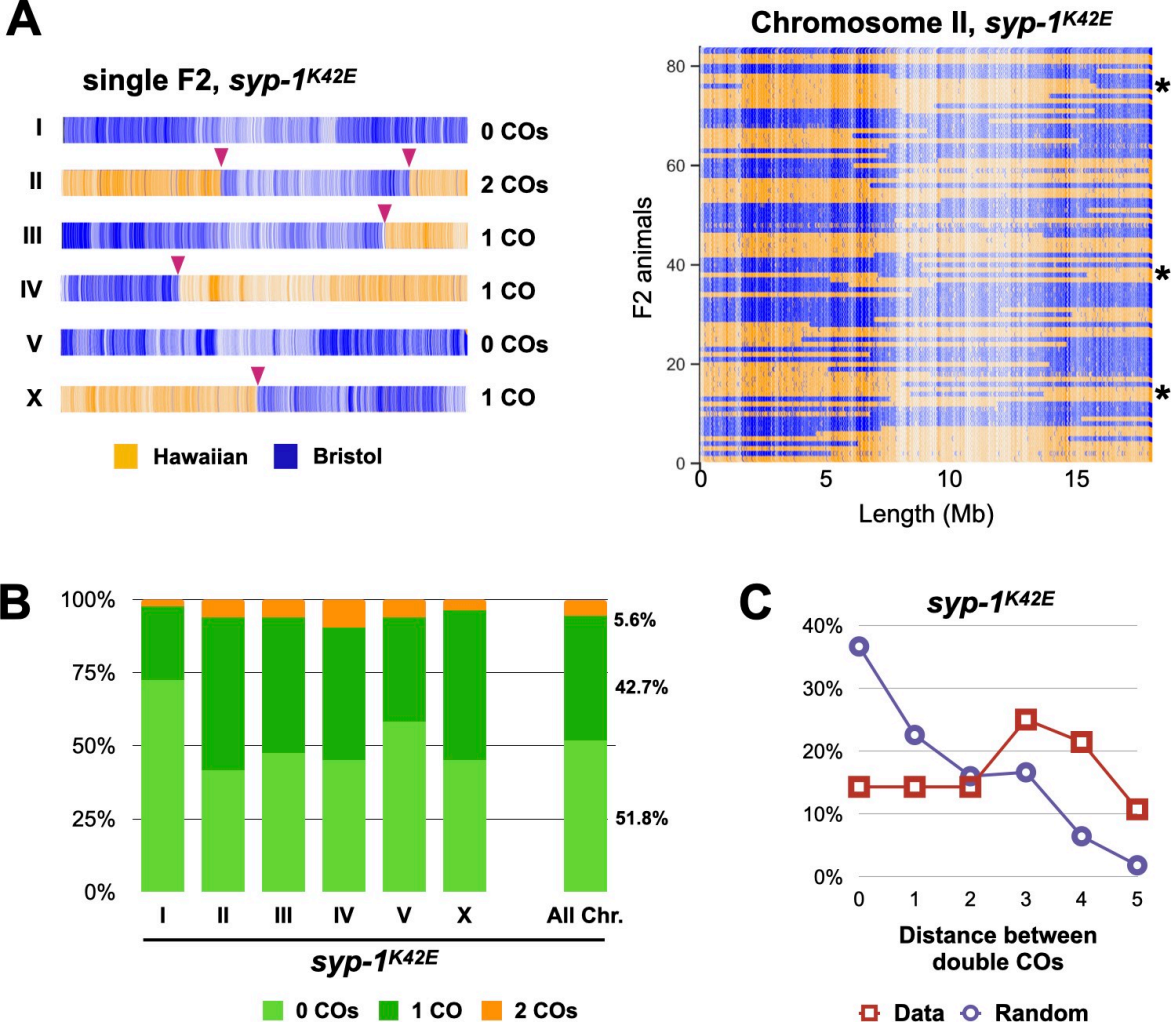
The N-terminus of SYP-1 regulates the number and distribution of crossovers. A) SNP profile of traceable meioses in *syp-1*^*K42E*^ (20°C) animals allowing localization of crossovers (COs). Each vertical line depicts a unique SNP (Hawaiian, yellow; Bristol, blue), while each horizontal bar represents a single chromosome. Left, SNP profile of a single cross-progeny showing chromosomes with single, double, and no crossovers (maroon arrows). Right, map of the SNPs along chromosome II in all 84 sequenced cross-progeny. Note the unique position of every crossover, and the presence of double crossovers in some progeny (several examples noted with asterisks). See S5 Fig for all mapped crossovers. B) Prevalence of crossovers for each chromosome and for all combined chromosomes in *syp-1*^*K42E*^ animals reveals the presence of double crossovers. C) Interference between crossovers is reduced but intact. In *syp-1*^*K42E*^ (20°C) chromosome that harbor two crossovers, crossovers are more distant from each other (maroon) than would be expected by chance (‘Random’, purple). The chromosomes were divided into 6 equal-sized intervals. N = 28 double crossovers from 84 F2 animals.

The presence of multiple GFP-COSA-1 foci along individual chromosomes in *syp-1*^*K42E*^ animals prompted us to measure crossover interference, which in *C*. *elegans* acts to ensure a single crossover (or GFP-COSA-1 focus) per homolog pair [4,40]. We found that GFP-COSA-1 foci in *syp-1*^*K42E*^ (20°C) animals are farther apart than would be expected if they were randomly distributed (Fig 5E), suggesting interference is still intact but acts on a shorter distance than in wild type. To ensure that crossover interference is indeed reduced in *syp-1*^*K42E*^ (20°C) worms, we identified the location of all crossovers genetically, by crossing *syp-1*^*K42E*^ worms that differ in thousands of SNPs and sequencing the genomes of their progeny (Figs 6A and 6B and S5). In *syp-1*^*K42E*^ animals 5.6% of chromosomes harbored double crossovers, higher than the 0% reported for wild-type animals [42]. The lower rate of genetic *versus* cytological double crossovers could be explained by the independent engagement of the four sisters, entailing that only one quarter of cytological double crossovers would manifest genetically. The median distance between double crossovers (14.14 Mb) was much greater than expected if the two crossovers were distributed randomly (3.17 Mb), indicating reduced but active crossover interference (Fig 6C), and corroborating our cytological data (Fig 5E).

Our sequencing data revealed additional features of the crossover landscape in *syp-1*^*K42E*^ (20°C) animals. First, chromosomes formed crossovers at variable rates, ranging from 27% for Chromosome I to 58% for Chromosome II (Fig 6B), which might reflect variable likelihood of zipping driven by different efficiencies of synapsis initiation on different chromosomes [2,32,34]. Second, while crossovers are generally suppressed in the middle of *C*. *elegans* chromosomes relative to the arms [43], crossovers in *syp-1*^*K42E*^ (20°C) animals were skewed toward the middle (S6A Fig). A similar effect was reported in mutants that, like *syp-1*^*K42E*^, fail to synapse all their chromosomes [44,45]. Finally, hypothesizing that forked chromosomes reflect zipping that fails to extend to the end of the chromosome, we tested whether crossovers are shifted toward the Pairing Centers, where SC assembly initiates [2,35]. Surprisingly, we did not observe a shift, but rather idiosyncratic effects on each arm (S6A Fig). We envisage that synapsis in *syp-1*^*K42E*^ (20°C) animals initiates at the Pairing Centers and that although forks are relatively stable over short timescales (30-60mins; S6B Fig), slower SC dynamics over the >24hrs nuclei spend in pachytene shift the zipped regions away from the Pairing Centers. Implicit in this idea is that the local chromosomal environment stabilizes the SC on some regions more than others, thereby facilitating crossover formation in those regions [21].

Altogether, our cytological and genetic analyses demonstrated that the N-terminus of SYP-1 plays a role in crossover regulation beyond zipping homologs together. The SC in both *syp-1*^*Δ8*^ and *syp-1*^*K42E*^ animals can successfully recruit pro-crossover factors, and, when the SC localizes between homologs, can also promote crossover formation. However, the SC in *syp-1*^*K42E*^ animals confers weaker crossover interference compared with the SC in wild-type and *syp-1*^*Δ8*^ animals.

## Discussion

Here we addressed structure-function relationships in the SC by combining recent advances in gene editing with the superior cytology and molecular genetics of the nematode *C*. *elegans*. We have employed a CRISPR/Cas9-based approach to generate and screen >50 mutants in a short, conserved stretch in one SC protein, SYP-1. These efforts yielded two new hypomorphs for SC functions, adding to a very short list of such mutants in worms. Detailed characterization revealed that while chromosome morphology is similar in the two mutants, underlying it are distinct alterations to the biophysical properties of the SC. One of these mutants also affected the distribution of crossovers between homologs.

Our analysis substantiates the recent understanding of the dual roles of the SC in both promoting and limiting crossovers [4]. In addition to bringing homologs together, pro-crossover factors are recruited to the SC, and in that way the SC limits crossovers to synapsed chromosomal regions. This idea receives support in this work, where we show that the SC that assembles on unpaired axes in *syp-1*^*K42E*^ (25°C) animals can recruit GFP-COSA-1 (see also [6,41]). Our work also strengthens the idea that, at least in *C*. *elegans*, the SC directly mediates crossover interference. Perturbations of the SC–including partial depletion of SYP-1, -2 and -3 and mutations in *syp-1*, *-4*, *-5* and *-6* ([4,11,13] and this work)–weaken crossover interference. The exact signal that spreads along entire chromosomes to mediate interference remains elusive, but several mechanisms have been suggested, including that diffusion within a liquid SC allows signals to be propagated [17]. Our work is consistent with this idea: the correlation between weaker crossover interference and more labile SC in *syp-1*^*K42E*^ animals (Figs 5 and 6) suggests that strong self-association of SC subunits is required for the spreading of the SC along chromosomes *and* for the spreading of an inhibitory signal that limits crossover number. Transient or permanent discontinuities along the SC in *syp-1*^*K42E*^ animals would block spreading of the SC when it assembles, and limit the spread of an inhibitory signal. Alternatively, the reduced self-association of SC subunits may alter the recruitment or the diffusion of a signaling molecule (“client protein”) that interacts with the SC, thereby altering signaling dynamics. Future high-resolution imaging efforts to reveal the internal organization of the SC in *syp-1*^*K42E*^ animals will help distinguish between these possibilities and shed light on the mechanisms of crossover interference.

**Fig 7:**
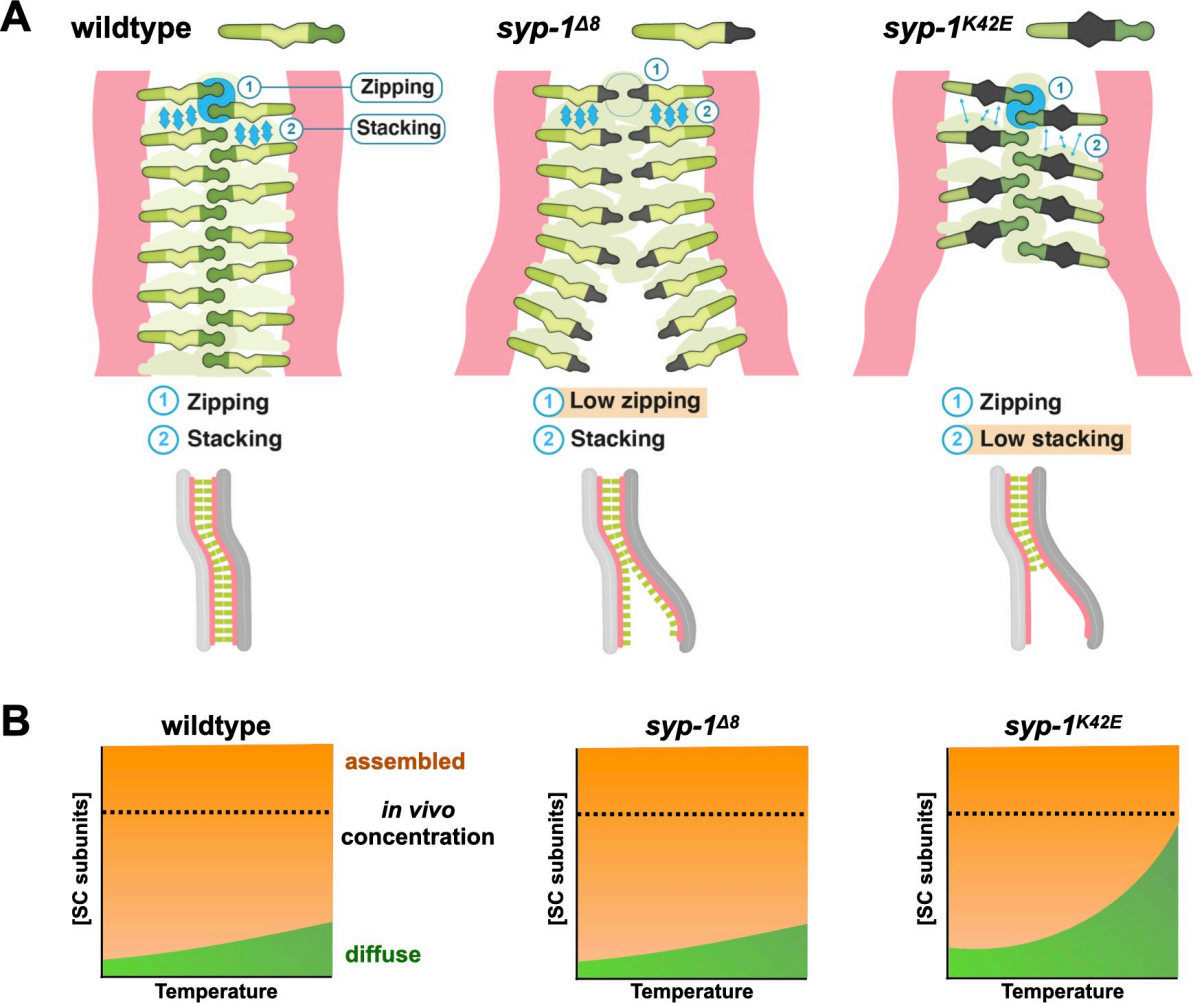
Model. A) Models of the two kinds of interactions allowing the SC to spread. Stacking interactions are shown as blue ovals, and zipping interactions as vertical arrows. Zipping interactions are weaker in *syp-1*^*Δ8*^ animals leading to inability to hold chromosomes together. Stacking interactions are weaker in *syp-1*^*K42E*^ animals, resulting in incomplete spreading of the SC along the chromosomes, and potential discontinuities within the SC. Bottom, resulting chromosome morphologies in each condition. Salmon, axis; green, SC. B) The weaker stacking interactions of the SC, shown as a phase diagram, where the concentration of the SC subunits is on the y-axis, and temperature is on the x-axis. Orange indicates conditions where SC material assembles, either onto chromosomes or into polycomplexes. In *syp-1*^*K42E*^ animals the phase diagram is shifted so that the SC fails assemble at 25°C with wild-type concentration of SC subunits. The high concentration of free SC subunits in those conditions results in spurious associations with unpaired axes.

Based on our analysis of SC mutants, we propose a model whereby the ability of the SC to zip chromosomes relies on two fundamental interactions (Fig 7A). The first interaction holds the two halves of the SC together (and by extension, the two axes and two homologs). We refer to it as a zipping interaction and propose that it arises from a high energetic cost of exposed surfaces in the middle of the SC. The second is a stacking interaction that allows the SC to recruit new subunits and spread laterally along chromosomes. Stacking interactions might reflect polymerization of SC subunits, or, alternatively, drive the phase separation of the SC [17]. In our model, zipping interactions are disrupted in *syp-1*^*Δ8*^ animals. As a result, the SC in *syp-1*^*Δ8*^ animals spread along the entire length of all chromosomes, but the chromosomes are not held together strongly and hence fork. In contrast, the SC in *syp-1*^*K42E*^ animals are mostly defective in stacking interactions, which aborts the zipping of the chromosomes, and leads to forks with asynapsed regions. As temperature increases, the stacking interactions diminish further (Fig 7B), resulting in high concentrations of free SC subunits, and consequently spurious associations with unpaired axes. The progressively weaker stacking interactions also manifest in the weaker tendency to form polycomplexes, the hypersensitivity to 1,6-hexanediol, and the reduced enrichment of the SC on chromosomes (Fig 4).

What are the molecular underpinnings of these interactions? The location of the SYP-1 N-terminus in the middle of the SC, the importance of N-terminal acetylation for SYP-1 function [30], and the conservation of charged residues revealed by our evolutionary analysis (S1A Fig), all point to a potential charge-charge interface that mediates zipping and disfavors association with unpaired axes. This interface likely overlaps with the amino acids deleted in *syp-1*^*Δ8*^, which include 4 charged residues. Stacking of SC subunits may likewise involve homo-oligomerization mediated by a charged interface that includes the lysine at position 42. However, we favor the idea that stacking interactions underlie the SC’s ability to phase separate, which can be driven by weak multivalent interactions [46]. For phase-separation of the SC, several non-mutually exclusive mechanisms have been proposed: weak hydrophobic interactions [17], interactions between the predicted coiled-coils, and interactions between charge-interacting elements (CIEs) [12]. The nature and location of the *syp-1*^*K42E*^ mutation–a charge-altering substitution outside the predicted coiled-coil–argues that hydrophobic interactions or the predicted coiled-coils are not solely responsible for phase separation. In contrast, *syp-1*^*K42E*^ alters an existing CIE, supporting a role for CIEs in driving phase separation of the SC [12,47]. Future biochemical characterization of protein-protein interactions in wild-type and mutant SC are likely to illuminate the molecular interactions that underlie SC organization.

## Materials and methods

### Worm strains and transgenes

All strains were grown at 20°C and cultured using standard methods [48]. Unless stated otherwise, all experiments were carried out at 20°C. The library of *syp-1* mutants, as well as *syp-1*^*K42E*^ and *syp-1*^*Δ8*^ in the Hawaiian background, were constructed using CRISPR/Cas9 ribonucleoprotein injection with a gRNA and repair template as described in S1B Fig. Mutants were then crossed out, and linkage between the mutations and the meiotic phenotype was verified. The temperature-sensitive *syp-1*^*K42E*^ was normally maintained at 15–20°C, and grown to adulthood at the indicated temperature to examine its meiotic phenotypes. *syp-1* mutant strains with fluorescently tagged axis and SC components (ROG211 and ROG 250) were maintained as balanced strains, since we detected potential genetic interaction with the GFP-SYP-3 transgene, that is itself a hypomorph [2]. *htp-3-wrmScarlet (slc1)* was constructed by CRISPR/Cas9 injection at the C-terminus of *htp-3* using *C*. *elegans*-optimized Scarlet protein [49]. For a full list of strains used see S1 Table. Progeny and male counts were performed by transferring an L4 to a new plate each day for three days, then counting progeny and males from each plate.

### CRISPR/Cas9 injections

L4 worms were grown for 24 hours at 20°C prior to injection. Injection mix was made in the following way: 1 μL *dpy-10* sgRNA (50 μM), 4 μL tRNA (200 μM), 3 μL guide RNA (200 μM) were incubated for 5 minutes at 95°C, then cooled to room temperature. 3.5 μL of the RNA solution was mixed with 3.5μL DNA template (1.5 μg/ml), 0.5μL Cas9 protein (IDT), and 0.5μL of DPY-10 template (200 μM). Dpy and Rol F1 progeny (positive for the co-injection mutation at *dpy-10*) were singled into plates, and their progeny analyzed.

### Immunofluorescence staining and mounting

Gonads were dissected and stained as in [50]. Briefly, 24hrs post-L4 adults were dissected in egg buffer (25 mM Hepes, pH 7.4, 118 mM NaCl, 48 mM KCl, 2 mM EDTA, 5 mM EGTA, 0.1% Tween-20, and 15 mM NaN_3_) and fixed in 1% formaldehyde before freeze cracking on dry ice. Dissected germlines were further fixed in methanol at −20°C for 1 min and rehydrated with PBST. Samples were then blocked with blocking reagent (Roche 01585762001) overnight at 4°C and incubated with primary antibodies overnight at 4°C. Primary antibodies were used at the following concentrations: anti-GFP (mouse, 1:1,000; Sigma F1804), anti-SYP-1 (goat, 1:500; [33]), anti-HTP-3 (guinea pig, 1:500; [11]), anti-HIM-8 (rat, 1:500; [31]), anti-ZHP-3 (rabbit, 1:10,000; [17]), anti-ZIM-3 (rabbit, 1:250; [34]), anti-H3K36me3 (rabbit, 1:1,000; Abcam), anti-DAO-5 (mouse monoclonal, 1:50; DSHB), GFP-booster-Atto488 nanobody (1:200; ChromoTek, Gba488-100). Appropriate secondary antibodies labeled with Alexa Fluor 488, Cy3, Cy5, or Alexa 647 were used at 1:500 dilution (Jackson Immunoresearch). Slides were mounted using fresh mounting media (450 μL NPG-glycerol, 35 μL 2M Tris, and 15 μL water). 1,6-hexanediol treatments were performed as previously described [17], and each gonad was scored as ‘dissolved’ if SC structures were indiscernible after treatment.

Detergent extraction (Figs 2C and 5C) was performed as described in [5]. Briefly, gonads were dissected in 30μl dissection buffer on an ethanol-washed 22×60mm coverslip. After dissection, dissection buffer was removed by pipetting leaving ~5μl on each slide. 50μl of spreading solution was added and gonads were gently spread across the coverslip using a pipet tip. Coverslips were left to dry at room temperature for 1 hour. Then, coverslips were moved to a 37°C heat block for 2 hours. Coverslips were washed in methanol for 20 min at −20°C and rehydrated by washing 3 times for 5 minutes in PBS-T, and immunostained as detailed above. Dissection buffer: 0.1% v/v Tween-20, 0.01% Tetramisole, v/v 85% HBSS. Fix: 4% w/v paraformaldehyde and 3.4% w/v sucrose in water. Spreading solution (for one coverslip): 32μl fix, 16μl 1% v/v Lipsol in water, 2μl Sarcosyl solution (1% w/v Sarcosyl in water).

### Genome wide mapping of crossovers

Hawaiian and Bristol strains differ by thousands of SNPs. *syp-1*^*K42E*^ Hawaiian males were crossed to *syp-1*^*K42E*^ Bristol hermaphrodites. F1s were isolated and backcrossed to *syp-1*^*K42E*^ Hawaiian males, and the F2s progeny of this cross were picked onto individual plates and grown until starved. Worms were maintained at 20°C throughout. Genomic DNA from the progeny of 84 F2 animals was isolated using Zymo DNA isolation kit, and was sequenced using a paired-end Nextera library preparation. NCBI accession: PRJNA699586.

The median depth coverage for each strain was between 5X and 11X where most strains had an 8X median coverage. Picard (a set of Java command line tools for manipulating high-throughput sequencing data) was used in order to align and determine the quality of sequencing reads. The sequencing data was first trimmed to eliminate the library barcode and the mitochondrial DNA reads were thrown out to give a data set of genomic reads without barcodes. GATK (genomics analysis toolkit) was used for variant calling (identifying SNPs) in the germline using a sequenced *Hawaiian* strain with the *syp-1*^*K42E*^ mutation and the most up to date *C*. *elegans* Bristol genome as reference genomes. Since the Hawaiian genome was deduced from our F2 progeny (rather than being sequenced directly), we manually decided crossover points based on how many SNPs were exchanged in a row and lack of background. For comparisons to the wild type recombination data (S6A Fig), we used the crossover rates and the designation of arms and middle of the chromosomes defined in [43]. To generate a ‘Random’ double crossover distribution (Fig 6C) we generated 2,100 double-crossovers by randomly pairing two mapped crossovers from a single chromosome. We repeated this analysis for all six chromosomes, weighing each chromosome according to the relative number of observed double crossovers.

### Irradiation and quantification of breaks

The number of DSBs induced by X-ray irradiation was calculated similarly to the method described in [40]. Briefly, *meIs8 [pie-1p*::*GFP*::*cosa-1 + unc-119(+)] II; pSUN-1*::*TIR-1*::*mRuby him-8(tm611) spo-11-aid IV* (ROG212) adult hermaphrodites were grown on 1 mM auxin (indole-3-acetic acid, VWR AAA10556-36) and therefore lacked SPO-11. These worms were subjected to increasing dosage of X-ray irradiation using a W-(ISOTOPE) source. 5 hours after irradiation the worms were dissected, and the number of COSA-1 foci in late pachytene nuclei, which mark COs, were counted. The results were modeled as in [40], with the exception of assuming a maximum number of 5 foci rather than 6, since the *him-8* mutation prevents X chromosome pairing and synapsis in our background. We solved to minimize the root mean square deviation, yielding a constant of c = 0.0028, meaning each 1000 rads of X-ray irradiation caused an average of 2.8 DSBs per homolog pair, or 1.4 DSBs per chromosome.

### Image acquisition and processing

Confocal microscopy images were collected as z-stacks (at 0.2μm intervals) using a 63x NA 1.40 objective on a Zeiss LSM 880 AiryScan microscopy system. Image processing and analysis was conducted using the ZEN software package (Blue 2.1). Partial maximum intensity projections are shown throughout. Foci were counted and confirmed using both maximum intensity projections as well as stepping through z-stacks. Transition zone length (Fig 3B) was calculated by measuring rows where most nuclei exhibited crescent-shaped chromatin. Meiotic entry was designated by the loading of HTP-3 onto chromosomes, and crescent-shaped was assessed based on chromatin morphology and the location of the nucleolus, marked by DAO-5. Pairing Centers foci (Fig 3C) separated by less than 0.3μm were considered as one focus. The polycomplex zone (Fig 4B) was calculated by measuring the portion of the gonad from the first nucleus with a polycomplex to the last row of nuclei with a polycomplex. Analysis of GFP-COSA-1 foci was performed on late pachytene nuclei.

Chromosome tracing, shown in Figs 2C and 5C and used for tracing GFP-COSA-1 to yield the data included in Fig 5B and 5E, was performed on 3D stacks using Imaris 9.2 (Bitplane). For Fig 5E, we normalized the chromosomal positions of 48 GFP-COSA-1 foci from fully synapsed chromosomes containing either one or two GFP-COSA-1 foci by mapping their position on a standard 7μm chromosome divided into 6 equal bins. For Fig 5E, we determined the interval for each focus and measured the distance between foci as ((Interval of 2nd focus)—(Interval of 1st focus)). To generate a ‘Random’ distribution, we randomly paired two foci to generate 24 double crossovers.

### Fluorescent protein quantification

For overall protein quantification (S2B Fig), animals expressing GFP-SYP-3 and HTP-3-wrmScarlet (ROG120, ROG211 and ROG250) were picked into 5μL of PEG glycerol (20% (v/v) PEG 20,000 and 20% (v/v) glycerol in PBS) and imaged immediately. The z-stacks were then summed to create a 2D image, and Fiji was used to quantify the individual fluorescent channels. Five late pachytene nuclei from each worm were measured and background fluorescence (outside the nuclei) was subtracted. For quantification of chromosome-associated versus nucleoplasmic SC (Fig 4D), live animals were immobilized and imaged as described in [2], and chromosome masks were calculated by using the ‘Surfaces’ tool in Imaris 9.2 (Bitplane). Chromosome-associated fluorescence was calculated by summing fluorescence intensity inside the masked region, and nucleoplasmic fluorescence was calculated by subtracting the chromosome-associated fluorescence from the total nuclear fluorescence. The values obtained for GFP-SYP-3 were normalized to the enrichment values obtained for HTP-3-wrmScarlet.

### Quantification of polycomplexes sphericity

Sphericity was assessed in Imaris 9.2 (Bitplane). 3D stacks were flattened in the z-dimension to eliminate aberrations caused by the poor axial resolution, the polycomplexes were automatically segmented using the ‘Surface’ tool, and the “Elipticity (oblate)” measurement was used.

### Alignment of Caenorhabditis species sequences

*Caenorhabditis syp-1* orthologues were identified by using *C*. *elegans* SYP-1 to query 10 *Caenorhabditis* species genomes using tBLASTn [51] implemented in WormBase or Caenorhabditis.org genome databases. All queries produced a single, high-confidence hit to an annotated gene. The coding sequences of *C*. *tropicalis* and *C*. *sinica* required manual editing of one conserved intron-exon boundary. Protein sequences were aligned using ClustalW [52] implemented in Geneious Prime 2019.0.4. Alignments were visualized and colored according to conservation in Jalview [53].

## Supporting information

S1 Fig Further characterization of SYP-1 conservation and mutagenesis strategy. A) Sequence alignment of SYP-1 protein sequences from species in the *elegans* group of the *Caenorhabditis* genus with percentages of amino acid conservation (darker blue indicates higher conservation) and predicted coiled-coil domains (dark grey lines below, based on the Paircoil2 program [54]). In addition to the first 13 a.a. in SYP-1, which are likely conserved due to N-acetylation ([30]; red line), and the phosphorylation site near the C-terminus ([28,29]; pink line), SYP-1^42−46^ is highly conserved. B) The N-terminus of SYP-1 in all of the elegans species group (species tree to the left) with the mutagenized domain highlighted by the red box. Below are the templates used to mutagenize this domain with the guide RNA shown above the PAM sequence in the templates. C) Schematic showing the steps to create the 19 mutants in SYP-1^42−46^ that were screened for meiotic defects. Following injection with a co-injection marker to generate ‘Roller’ animals, heterozygous worms were identified by PCR, restriction enzyme digestion and sequencing, and finally isolating homozygous worms that were not sterile.
(TIF)

S2 Fig Further characterization of the effect of *syp-1* mutants on the SC. A) The SC is associated with the unpaired X chromosomes in pachytene nuclei from *him-8 syp-1*^*Δ8*^ worms. The SC is stained with antibodies against the SC (anti-SYP-1; green) and the histone mark H3K36me3 (magenta), which marks chromatin on the autosomes [55]. The X chromosomes, lacking H3K36me3, are marked with blue arrowheads. Scale bar = 5μm. B) Ratio of the fluorescence of GFP-SYP-3 and HTP-3-wrmScarlet in the indicated conditions, relative to the average values in wild-type animals. The bars indicate the median fluorescence, and the box indicate the middle 50%. N>20 nuclei for each condition. Significant comparisons to wild type are shown (Student’s t-test, p<0.05). While the differences are statistically significant, the overall reduction in the protein levels of SC subunits is much smaller that the 60–70% reduction previously shown to affect SC assembly or crossover interference [4,32].
(TIF)

S3 Fig Further characterization of polycomplexes in *syp-1* mutants. A) Sphericity measurements of polycomplexes from the indicated genotypes showing that *htp-3 syp-1*^*Δ8*^ has less spherical polycomplexes compared with *syp-1(+)* polycomplexes (related to Fig 4A and 4B). The bars indicate the median sphericity measurement of all polycomplexes from each strain, and the boxes indicate the middle 50%. N>80 polycomplexes from at least 3 gonads for each genotype. Significant pairwise comparisons are indicated (Student’s t-test, P<0.05). See Methods for more details. B) Polycomplexes in *syp-1* mutants are still able to correctly re-localize ZHP-3. In late pachytene, concomitant with the increasing number of polycomplexes per nucleus, ZHP-3 changes its localization from spreading over entire polycomplexes to small foci abutting polycomplexes [17]. This is also observed in *syp-1* mutants. Immunofluorescence images of the indicated genotypes were stained with anti-SYP-1 antibodies (polycomplexes, red), anti-ZHP-3 antibodies (green), and DAPI (DNA, blue). Right, highlighted nuclei showing both complete overlapping of ZHP-3 and polycomplexes (top), and localization into foci (bottom). Scale bar = 3μm.
(TIF)

S4 Fig Empirical determination of the number of breaks generated per dose of X-ray irradiation. A) Animals that cannot make endogenous breaks or pair the X chromosome (*him-8 spo-11(-); GFP-COSA-1*) were subjected to varying doses of X-ray irradiation. In each condition, the number of GFP-COSA-1 foci per nuclei is plotted. As expected, the number plateaus at an average of 5 GFP-COSA-1 foci, since only the 5 autosomes can undergo a crossover. B) The average number of the observed GFP-COSA-1 foci are shown as blue dots. The best fitting curve (red line) was calculated as in [40], and was found to result from a conversion factor c = 0.0028, meaning each 1 Rad of X-ray irradiation is causing an average of 0.0028 breaks per homolog pair.
(TIF)

S5 Fig Map of all crossovers detected in *syp-1^K42E^* animals. Graphs of all crossovers identified among the 84 *syp-1*^*K42E*^ (20°C) F2 progeny analyzed. Each black dot represents a crossover event, and each horizonal line represents a double crossover event. The x-axis is the location along each of the six chromosomes. Note the relatively wide spacing of double-crossover events (summarized in Fig 6C).
(TIF)

S6 Fig Further characterization of crossovers in *syp-1^K42E^* animals. A) Crossovers in *syp-1*^*K42E*^ (20°C) animals are shifted towards the middle of the chromosome, but not toward the Pairing Center end. Crossover rate (calculated in centi-Morgan [cM] per Mb) was calculated for each arm and middle of all chromosomes, as defined by [43]. Ratio between the values measured for *syp-1*^*K42E*^ (20°C) animals and those measured for wild-type animals [43] are shown, with each data point representing a chromosome or a chromosome arm. Note the more than 2-fold higher crossover rate in the middle of the chromosomes, and the lower, and much more varied rate on the arms, with no enrichment on the arms harboring the Pairing Centers. B) Selected images from a time-lapse series of a live *syp1*^*Δ8*^ *GFP-SYP-3* and *syp1*^*K42E*^ *GFP-SYP-3* worms. The forked chromosomes (blue arrowheads) persists throughout the duration of the imaging (30–60 minutes). Note that in the presence of the GFP-SYP-3 transgene, the SC localizes to both the paired and unpaired regions on forked chromosomes in *syp1*^*K42E*^ (20°C) mutants. Times are shown as h:mm:ss. Single z-slices are shown for clarity. Scale bars = 3μm.
(TIF)

S1 Table Worm strains used in this study. List of all worm strains used in this study and their sources.
(PDF)

## References

[1] Rog O. and Dernburg A.F., Chromosome pairing and synapsis during Caenorhabditis elegans meiosis. Curr Opin Cell Biol, 2013. 25(3): p. 349–56. PMC3694717 10.1016/j.ceb.2013.03.003 23578368 PMC3694717

[2] Rog O. and Dernburg A.F., Direct Visualization Reveals Kinetics of Meiotic Chromosome Synapsis. Cell Rep, 2015. PMC4565782 10.1016/j.celrep.2015.02.032 25772351 PMC4565782

[3] Page S.L. and Hawley R.S., The genetics and molecular biology of the synaptonemal complex. Annu Rev Cell Dev Biol, 2004. 20: p. 525–58. 10.1146/annurev.cellbio.19.111301.155141 15473851

[4] Libuda D.E., et al., Meiotic chromosome structures constrain and respond to designation of crossover sites. Nature, 2013. 502(7473): p. 703–6. PMC3920622 10.1038/nature12577 24107990 PMC3920622

[5] Woglar A. and Villeneuve A.M., Dynamic Architecture of DNA Repair Complexes and the Synaptonemal Complex at Sites of Meiotic Recombination. Cell, 2018. 173(7): p. 1678–1691 e16. PMC6003859 10.1016/j.cell.2018.03.066 29754818 PMC6003859

[6] Cahoon C.K., Helm J.M., and Libuda D.E., Synaptonemal Complex Central Region Proteins Promote Localization of Pro-crossover Factors to Recombination Events During Caenorhabditis elegans Meiosis. Genetics, 2019. 213(2): p. 395–409. PMC6781886 10.1534/genetics.119.302625 31431470 PMC6781886

[7] MacQueen A.J., et al., Synapsis-dependent and -independent mechanisms stabilize homolog pairing during meiotic prophase in C. elegans. Genes Dev, 2002. 16(18): p. 2428–42. PMC187442 10.1101/gad.1011602 12231631 PMC187442

[8] Colaiacovo M.P., et al., Synaptonemal complex assembly in C. elegans is dispensable for loading strand-exchange proteins but critical for proper completion of recombination. Dev Cell, 2003. 5(3): p. 463–74. 10.1016/s1534-5807(03)00232-6 12967565

[9] Smolikov S., Schild-Prufert K., and Colaiacovo M.P., A yeast two-hybrid screen for SYP-3 interactors identifies SYP-4, a component required for synaptonemal complex assembly and chiasma formation in Caenorhabditis elegans meiosis. PLoS Genet, 2009. 5(10): p. e1000669. PMC2742731 10.1371/journal.pgen.1000669 19798442 PMC2742731

[10] Smolikov S., et al., SYP-3 restricts synaptonemal complex assembly to bridge paired chromosome axes during meiosis in Caenorhabditis elegans. Genetics, 2007. 176(4): p. 2015–25. PMC1950610 10.1534/genetics.107.072413 17565948 PMC1950610

[11] Hurlock M.E., et al., Identification of novel synaptonemal complex components in C. elegans. J Cell Biol, 2020. 219(5). PMC7199856 10.1083/jcb.201910043 32211899 PMC7199856

[12] Zhang Z., et al., Multivalent weak interactions between assembly units drive synaptonemal complex formation. J Cell Biol, 2020. 219(5). PMC7199860 10.1083/jcb.201910086 32211900 PMC7199860

[13] Köhler S., et al., The interaction of crossover formation and the dynamic architecture of the synaptonemal complex during meiosis. bioRxiv, 2020: p. 2020.02.16.947804.

[14] Schucker K., et al., Elucidation of synaptonemal complex organization by super-resolution imaging with isotropic resolution. Proc Natl Acad Sci U S A, 2015. 112(7): p. 2029–33. PMC4343094 10.1073/pnas.1414814112 25646409 PMC4343094

[15] Schild-Prufert K., et al., Organization of the synaptonemal complex during meiosis in Caenorhabditis elegans. Genetics, 2011. 189(2): p. 411–21. PMC3189812 10.1534/genetics.111.132431 21840865 PMC3189812

[16] Dunce J.M., et al., Structural basis of meiotic chromosome synapsis through SYCP1 self-assembly. Nat Struct Mol Biol, 2018. 25(7): p. 557–569. PMC6606445 10.1038/s41594-018-0078-9 29915389 PMC6606445

[17] Rog O., Kohler S., and Dernburg A.F., The synaptonemal complex has liquid crystalline properties and spatially regulates meiotic recombination factors. Elife, 2017. 6. PMC5268736 10.7554/eLife.21455 28045371 PMC5268736

[18] Pattabiraman D., et al., Meiotic recombination modulates the structure and dynamics of the synaptonemal complex during C. elegans meiosis. PLoS Genet, 2017. 13(3): p. e1006670. PMC5384771 10.1371/journal.pgen.1006670 28339470 PMC5384771

[19] Sym M. and Roeder G.S., Zip1-induced changes in synaptonemal complex structure and polycomplex assembly. J Cell Biol, 1995. 128(4): p. 455–66. PMC2199901 10.1083/jcb.128.4.455 7860625 PMC2199901

[20] Ollinger R., Alsheimer M., and Benavente R., Mammalian protein SCP1 forms synaptonemal complex-like structures in the absence of meiotic chromosomes. Mol Biol Cell, 2005. 16(1): p. 212–7. PMC539165 10.1091/mbc.e04-09-0771 15496453 PMC539165

[21] Billmyre K.K., et al., X chromosome and autosomal recombination are differentially sensitive to disruptions in SC maintenance. Proc Natl Acad Sci U S A, 2019. 116(43): p. 21641–21650. PMC6815145 10.1073/pnas.1910840116 31570610 PMC6815145

[22] Jeffress J.K., et al., The formation of the central element of the synaptonemal complex may occur by multiple mechanisms: the roles of the N- and C-terminal domains of the Drosophila C(3)G protein in mediating synapsis and recombination. Genetics, 2007. 177(4): p. 2445–56. PMC2219479 10.1534/genetics.107.078717 17947423 PMC2219479

[23] Tung K.S. and Roeder G.S., Meiotic chromosome morphology and behavior in zip1 mutants of Saccharomyces cerevisiae. Genetics, 1998. 149(2): p. 817–32. PMC1460213 9611194 10.1093/genetics/149.2.817 PMC1460213

[24] Voelkel-Meiman K., et al., Synaptonemal Complex Proteins of Budding Yeast Define Reciprocal Roles in MutSgamma-Mediated Crossover Formation. Genetics, 2016. 203(3): p. 1091–103. PMC4937465 10.1534/genetics.115.182923 27184389 PMC4937465

[25] Voelkel-Meiman K., et al., Crossover recombination and synapsis are linked by adjacent regions within the N terminus of the Zip1 synaptonemal complex protein. PLoS Genet, 2019. 15(6): p. e1008201. PMC6605668 10.1371/journal.pgen.1008201 31220082 PMC6605668

[26] Roelens B., et al., Spatial Regulation of Polo-Like Kinase Activity During Caenorhabditis elegans Meiosis by the Nucleoplasmic HAL-2/HAL-3 Complex. Genetics, 2019. 213(1): p. 79–96. PMC6727811 10.1534/genetics.119.302479 31345995 PMC6727811

[27] Zhang W., et al., HAL-2 promotes homologous pairing during Caenorhabditis elegans meiosis by antagonizing inhibitory effects of synaptonemal complex precursors. PLoS Genet, 2012. 8(8): p. e1002880. PMC3415444 10.1371/journal.pgen.1002880 22912597 PMC3415444

[28] Sato-Carlton A., et al., Phosphorylation of the synaptonemal complex protein SYP-1 promotes meiotic chromosome segregation. J Cell Biol, 2018. 217(2): p. 555–570. PMC5800814 10.1083/jcb.201707161 29222184 PMC5800814

[29] Garcia-Muse T., et al., A Meiotic Checkpoint Alters Repair Partner Bias to Permit Inter-sister Repair of Persistent DSBs. Cell Rep, 2019. 26(3): p. 775–787 e5. PMC6334227 10.1016/j.celrep.2018.12.074 30650366 PMC6334227

[30] Gao J., et al., N-terminal acetylation promotes synaptonemal complex assembly in C. elegans. Genes Dev, 2016. 30(21): p. 2404–2416. PMC5131780 10.1101/gad.277350.116 27881602 PMC5131780

[31] Phillips C.M., et al., HIM-8 binds to the X chromosome pairing center and mediates chromosome-specific meiotic synapsis. Cell, 2005. 123(6): p. 1051–63. PMC4435792 10.1016/j.cell.2005.09.035 16360035 PMC4435792

[32] Hayashi M., Mlynarczyk-Evans S., and Villeneuve A.M., The synaptonemal complex shapes the crossover landscape through cooperative assembly, crossover promotion and crossover inhibition during Caenorhabditis elegans meiosis. Genetics, 2010. 186(1): p. 45–58. PMC2940310 10.1534/genetics.110.115501 20592266 PMC2940310

[33] Harper N.C., et al., Pairing centers recruit a Polo-like kinase to orchestrate meiotic chromosome dynamics in C. elegans. Dev Cell, 2011. 21(5): p. 934–47. PMC4343031 10.1016/j.devcel.2011.09.001 22018922 PMC4343031

[34] Phillips C.M. and Dernburg A.F., A family of zinc-finger proteins is required for chromosome-specific pairing and synapsis during meiosis in C. elegans. Dev Cell, 2006. 11(6): p. 817–29. 10.1016/j.devcel.2006.09.020 17141157

[35] MacQueen A.J., et al., Chromosome sites play dual roles to establish homologous synapsis during meiosis in C. elegans. Cell, 2005. 123(6): p. 1037–50. PMC4435800 10.1016/j.cell.2005.09.034 16360034 PMC4435800

[36] Hughes S.E., et al., The E3 ubiquitin ligase Sina regulates the assembly and disassembly of the synaptonemal complex in Drosophila females. PLoS Genet, 2019. 15(5): p. e1008161. PMC6544331 10.1371/journal.pgen.1008161 31107865 PMC6544331

[37] Goodyer W., et al., HTP-3 links DSB formation with homolog pairing and crossing over during C. elegans meiosis. Dev Cell, 2008. 14(2): p. 263–74. 10.1016/j.devcel.2007.11.016 18267094

[38] Couteau F., et al., A component of C. elegans meiotic chromosome axes at the interface of homolog alignment, synapsis, nuclear reorganization, and recombination. Curr Biol, 2004. 14(7): p. 585–92. 10.1016/j.cub.2004.03.033 15062099

[39] Wang K., et al., Increasing the Genetic Recombination Frequency by Partial Loss of Function of the Synaptonemal Complex in Rice. Mol Plant, 2015. 8(8): p. 1295–8. 10.1016/j.molp.2015.04.011 25936677

[40] Yokoo R., et al., COSA-1 reveals robust homeostasis and separable licensing and reinforcement steps governing meiotic crossovers. Cell, 2012. 149(1): p. 75–87. PMC3339199 10.1016/j.cell.2012.01.052 22464324 PMC3339199

[41] Almanzar D.E., Gordon S.G., and Rog O., Meiotic sister chromatid exchanges are rare in C. elegans. bioRxiv, 2020: p. 2020.07.22.216614. 10.1016/j.cub.2020.11.018 PMC8051885 33740426

[42] Tsai C.J., et al., Meiotic crossover number and distribution are regulated by a dosage compensation protein that resembles a condensin subunit. Genes Dev, 2008. 22(2): p. 194–211. PMC2192754 10.1101/gad.1618508 18198337 PMC2192754

[43] Rockman M.V. and Kruglyak L., Recombinational landscape and population genomics of Caenorhabditis elegans. PLoS Genet, 2009. 5(3): p. e1000419. PMC2652117 10.1371/journal.pgen.1000419 19283065 PMC2652117

[44] Carlton P.M., Farruggio A.P., and Dernburg A.F., A link between meiotic prophase progression and crossover control. PLoS Genet, 2006. 2(2): p. e12. PMC1359072 APF performed the experiments. PMC, APF, and AFD analyzed the data. PMC and AFD wrote the paper. 10.1371/journal.pgen.0020012 16462941 PMC1359072

[45] Li Q., et al., The tumor suppressor BRCA1-BARD1 complex localizes to the synaptonemal complex and regulates recombination under meiotic dysfunction in Caenorhabditis elegans. PLoS Genet, 2018. 14(11): p. e1007701. PMC6211623 10.1371/journal.pgen.1007701 30383767 PMC6211623

[46] Alberti S., Gladfelter A., and Mittag T., Considerations and Challenges in Studying Liquid-Liquid Phase Separation and Biomolecular Condensates. Cell, 2019. 176(3): p. 419–434. PMC6445271 10.1016/j.cell.2018.12.035 30682370 PMC6445271

[47] Pak C.W., et al., Sequence Determinants of Intracellular Phase Separation by Complex Coacervation of a Disordered Protein. Mol Cell, 2016. 63(1): p. 72–85. PMC4973464 10.1016/j.molcel.2016.05.042 27392146 PMC4973464

[48] Brenner S., The genetics of Caenorhabditis elegans. Genetics, 1974. 77(1): p. 71–94. PMC1213120 4366476 10.1093/genetics/77.1.71 PMC1213120

[49] El Mouridi S., et al., Reliable CRISPR/Cas9 Genome Engineering in Caenorhabditis elegans Using a Single Efficient sgRNA and an Easily Recognizable Phenotype. G3 (Bethesda), 2017. 7(5): p. 1429–1437. PMC5427500 10.1534/g3.117.040824 28280211 PMC5427500

[50] Phillips C.M., McDonald K.L., and Dernburg A.F., Cytological analysis of meiosis in Caenorhabditis elegans. Methods Mol Biol, 2009. 558: p. 171–95. PMC3644504 10.1007/978-1-60761-103-5_11 19685325 PMC3644504

[51] Altschul S.F., et al., Gapped BLAST and PSI-BLAST: a new generation of protein database search programs. Nucleic Acids Res, 1997. 25(17): p. 3389–402. PMC146917 10.1093/nar/25.17.3389 9254694 PMC146917

[52] Larkin M.A., et al., Clustal W and Clustal X version 2.0. Bioinformatics, 2007. 23(21): p. 2947–8. 10.1093/bioinformatics/btm404 17846036

[53] Clamp M., et al., The Jalview Java alignment editor. Bioinformatics, 2004. 20(3): p. 426–7. 10.1093/bioinformatics/btg430 14960472

[54] McDonnell A.V., et al., Paircoil2: improved prediction of coiled coils from sequence. Bioinformatics, 2006. 22(3): p. 356–8. 10.1093/bioinformatics/bti797 16317077

[55] Kreher J., et al., Distinct Roles of Two Histone Methyltransferases in Transmitting H3K36me3-Based Epigenetic Memory Across Generations in Caenorhabditis elegans. Genetics, 2018. 210(3): p. 969–982. PMC6218224. 10.1534/genetics.118.301353 30217796 PMC6218224

