# Supplementary figures and images for: Synaptonemal Complex dimerization regulates chromosome alignment and crossover patterning in meiosis
Source: PLoS Genet. 2021 Mar 17;17(3):e1009205. doi: 10.1371/journal.pgen.1009205 (PMC7968687; doi:10.1371/journal.pgen.1009205)

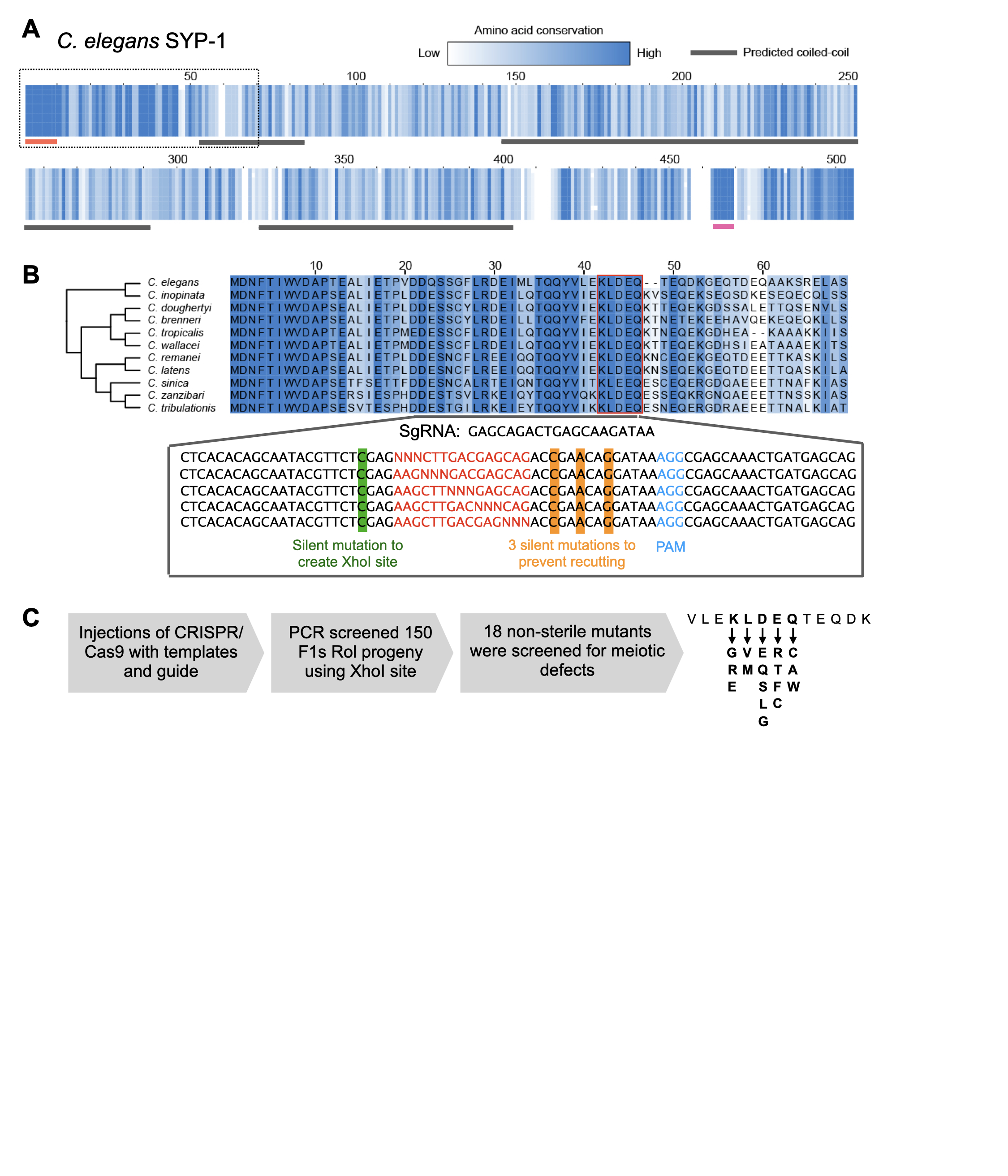

Supplement: S1 Fig — A) Sequence alignment of SYP-1 protein sequences from species in the elegans group of the Caenorhabditis genus with percentages of amino acid conservation (darker blue indicates higher conservation) and predicted coiled-coil domains (dark grey lines below, based on the Paircoil2 program [54]). In addition to the first 13 a.a. in SYP-1, which are likely conserved due to N-acetylation ([30]; red line), and the phosphorylation site near the C-terminus ([28,29]; pink line), SYP-142−46 is highly conserved. B) The N-terminus of SYP-1 in all of the elegans species group (species tree to the left) with the mutagenized domain highlighted by the red box. Below are the templates used to mutagenize this domain with the guide RNA shown above the PAM sequence in the templates. C) Schematic showing the steps to create the 19 mutants in SYP-142−46 that were screened for meiotic defects. Following injection with a co-injection marker to generate ‘Roller’ animals, heterozygous worms were identified by PCR, restriction enzyme digestion and sequencing, and finally isolating homozygous worms that were not sterile. (TIF) [file pgen.1009205.s001.tif]

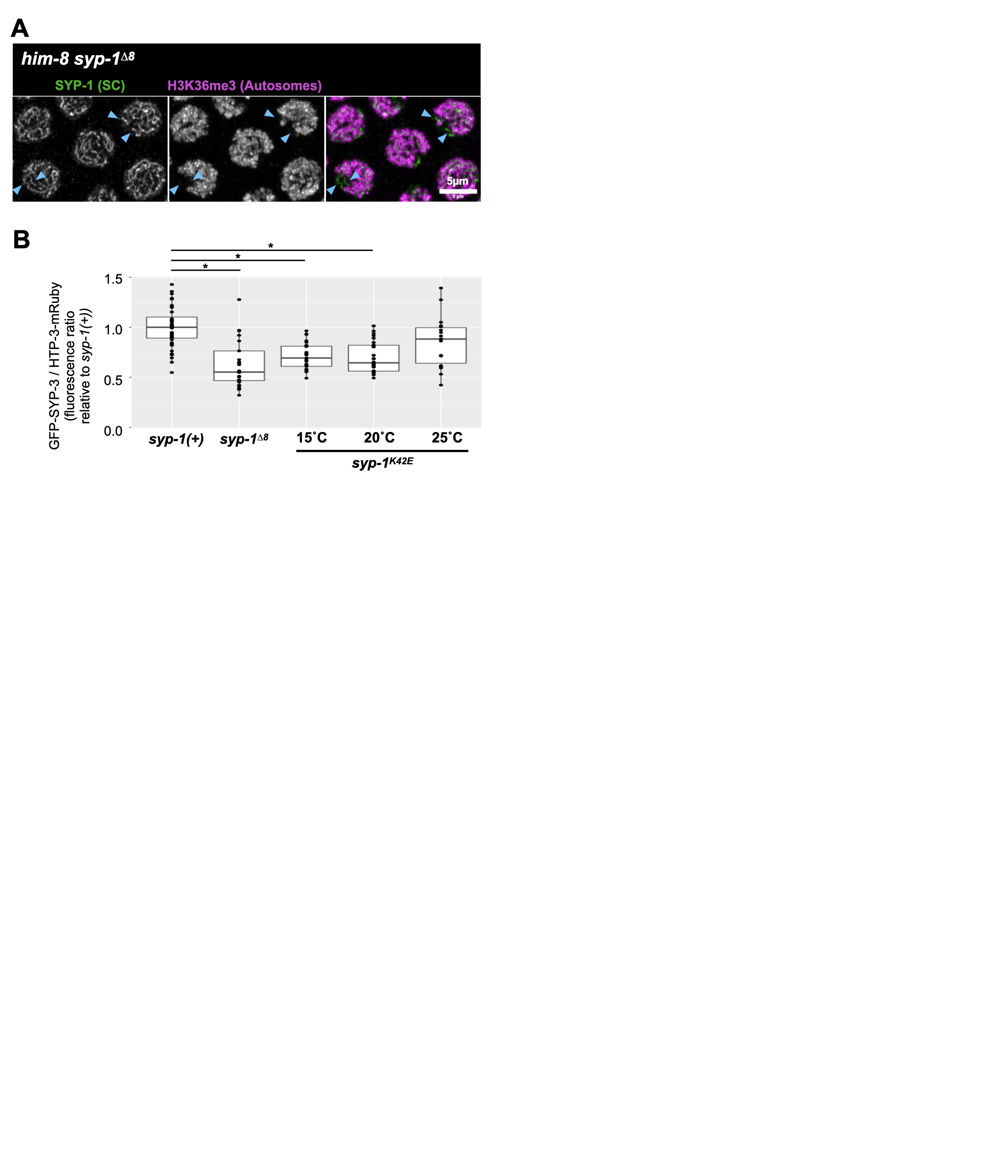

Supplement: S2 Fig — A) The SC is associated with the unpaired X chromosomes in pachytene nuclei from him-8 syp-1Δ8 worms. The SC is stained with antibodies against the SC (anti-SYP-1; green) and the histone mark H3K36me3 (magenta), which marks chromatin on the autosomes [55]. The X chromosomes, lacking H3K36me3, are marked with blue arrowheads. Scale bar = 5μm. B) Ratio of the fluorescence of GFP-SYP-3 and HTP-3-wrmScarlet in the indicated conditions, relative to the average values in wild-type animals. The bars indicate the median fluorescence, and the box indicate the middle 50%. N>20 nuclei for each condition. Significant comparisons to wild type are shown (Student’s t-test, p<0.05). While the differences are statistically significant, the overall reduction in the protein levels of SC subunits is much smaller that the 60–70% reduction previously shown to affect SC assembly or crossover interference [4,32]. (TIF) [file pgen.1009205.s002.tif]

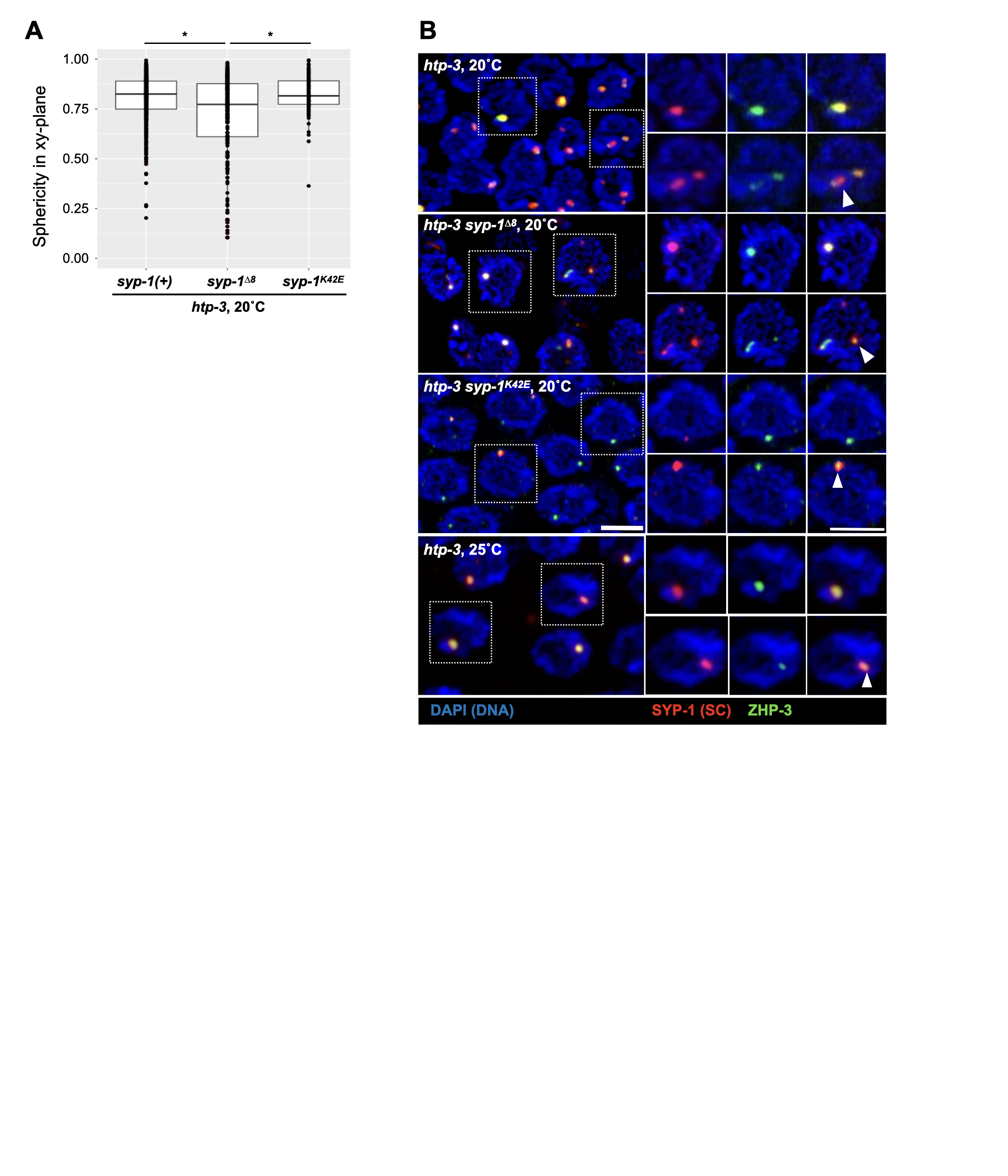

Supplement: S3 Fig — A) Sphericity measurements of polycomplexes from the indicated genotypes showing that htp-3 syp-1Δ8 has less spherical polycomplexes compared with syp-1(+) polycomplexes (related to Fig 4A and 4B). The bars indicate the median sphericity measurement of all polycomplexes from each strain, and the boxes indicate the middle 50%. N>80 polycomplexes from at least 3 gonads for each genotype. Significant pairwise comparisons are indicated (Student’s t-test, P<0.05). See Methods for more details. B) Polycomplexes in syp-1 mutants are still able to correctly re-localize ZHP-3. In late pachytene, concomitant with the increasing number of polycomplexes per nucleus, ZHP-3 changes its localization from spreading over entire polycomplexes to small foci abutting polycomplexes [17]. This is also observed in syp-1 mutants. Immunofluorescence images of the indicated genotypes were stained with anti-SYP-1 antibodies (polycomplexes, red), anti-ZHP-3 antibodies (green), and DAPI (DNA, blue). Right, highlighted nuclei showing both complete overlapping of ZHP-3 and polycomplexes (top), and localization into foci (bottom). Scale bar = 3μm. (TIF) [file pgen.1009205.s003.tif]

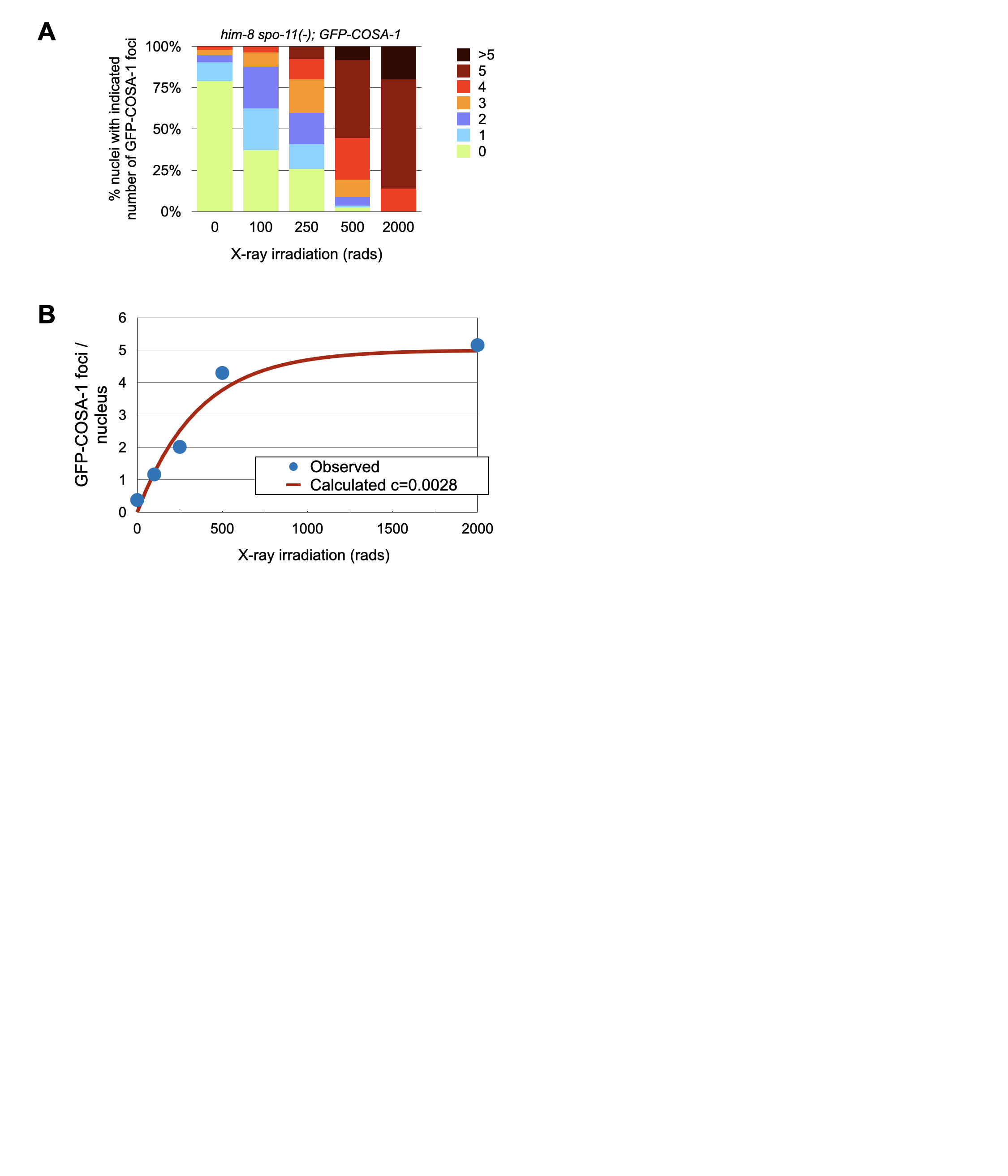

Supplement: S4 Fig — A) Animals that cannot make endogenous breaks or pair the X chromosome (him-8 spo-11(-); GFP-COSA-1) were subjected to varying doses of X-ray irradiation. In each condition, the number of GFP-COSA-1 foci per nuclei is plotted. As expected, the number plateaus at an average of 5 GFP-COSA-1 foci, since only the 5 autosomes can undergo a crossover. B) The average number of the observed GFP-COSA-1 foci are shown as blue dots. The best fitting curve (red line) was calculated as in [40], and was found to result from a conversion factor c = 0.0028, meaning each 1 Rad of X-ray irradiation is causing an average of 0.0028 breaks per homolog pair. (TIF) [file pgen.1009205.s004.tif]

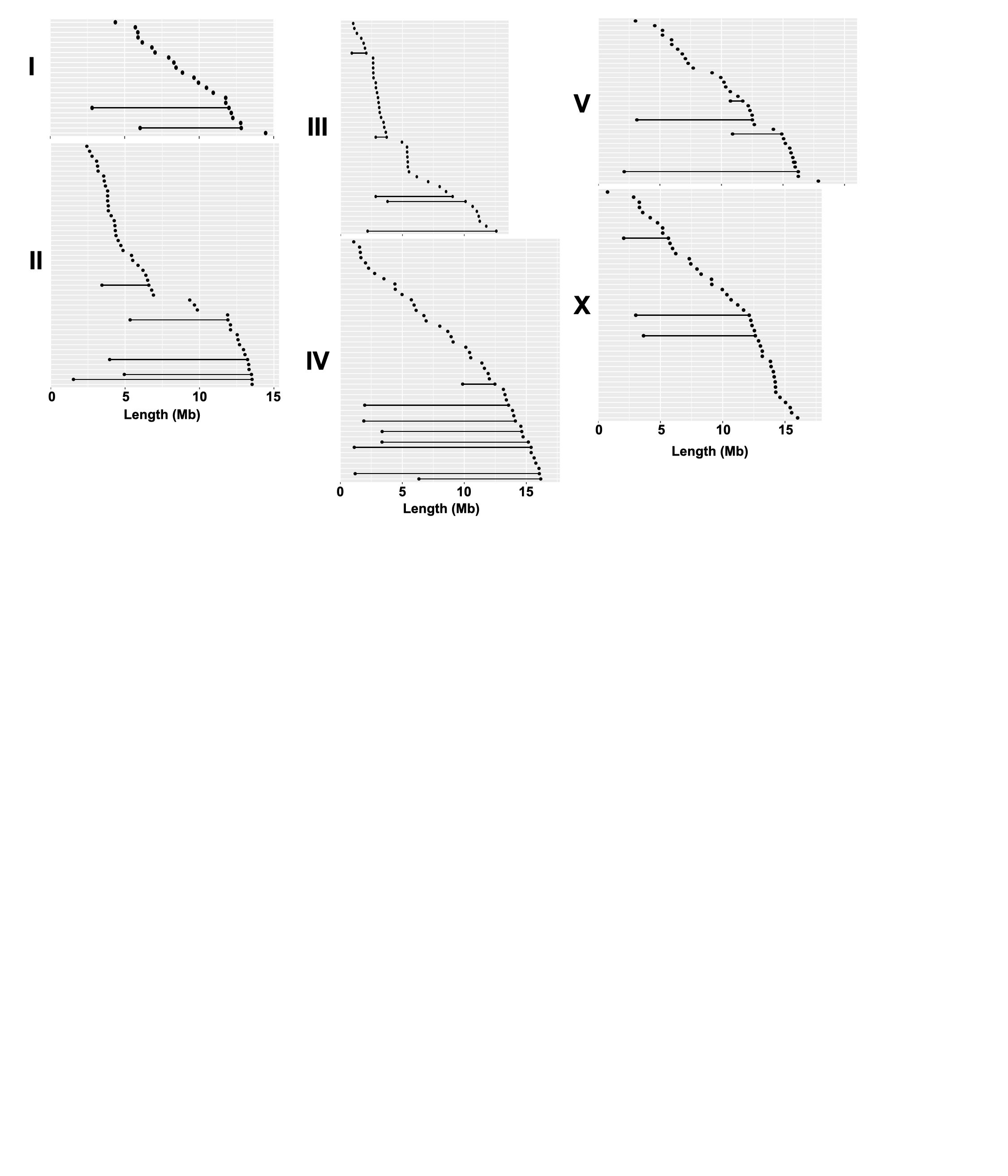

Supplement: S5 Fig — Graphs of all crossovers identified among the 84 syp-1K42E (20°C) F2 progeny analyzed. Each black dot represents a crossover event, and each horizonal line represents a double crossover event. The x-axis is the location along each of the six chromosomes. Note the relatively wide spacing of double-crossover events (summarized in Fig 6C). (TIF) [file pgen.1009205.s005.tif]

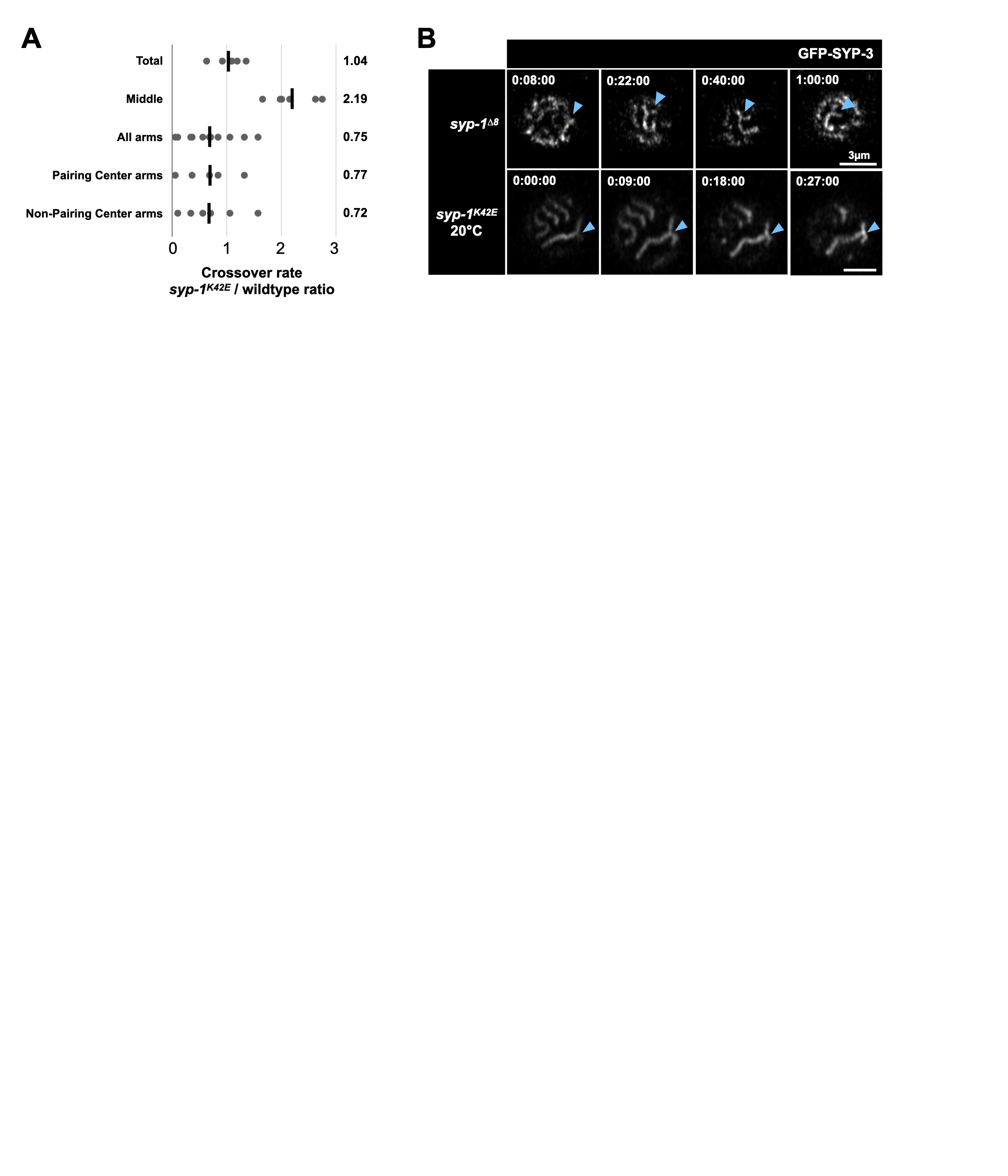

Supplement: S6 Fig — A) Crossovers in syp-1K42E (20°C) animals are shifted towards the middle of the chromosome, but not toward the Pairing Center end. Crossover rate (calculated in centi-Morgan [cM] per Mb) was calculated for each arm and middle of all chromosomes, as defined by [43]. Ratio between the values measured for syp-1K42E (20°C) animals and those measured for wild-type animals [43] are shown, with each data point representing a chromosome or a chromosome arm. Note the more than 2-fold higher crossover rate in the middle of the chromosomes, and the lower, and much more varied rate on the arms, with no enrichment on the arms harboring the Pairing Centers. B) Selected images from a time-lapse series of a live syp1Δ8 GFP-SYP-3 and syp1K42E GFP-SYP-3 worms. The forked chromosomes (blue arrowheads) persists throughout the duration of the imaging (30–60 minutes). Note that in the presence of the GFP-SYP-3 transgene, the SC localizes to both the paired and unpaired regions on forked chromosomes in syp1K42E (20°C) mutants. Times are shown as h:mm:ss. Single z-slices are shown for clarity. Scale bars = 3μm. (TIF) [file pgen.1009205.s006.tif]
