# Supplementary material for: Synaptonemal Complex dimerization regulates chromosome alignment and crossover patterning in meiosis
Source: PLoS Genet. 2021 Mar 17;17(3):e1009205. doi: 10.1371/journal.pgen.1009205 (PMC7968687; doi:10.1371/journal.pgen.1009205)
Supplement: S1 Table — List of all worm strains used in this study and their sources. (PDF) [file pgen.1009205.s007.pdf]

S1 Table. Worm strains used in this study

Strain	Genotype	Source/Construction
N2	Bristol wild type	CGC
CB4856	Hawaiian wild type isolate	CGC
CA257	*him-8 (tm611) IV*	CGC
TY5038	*htp-3 (tm3656) I/hT2 [bli-4 (e937) let-? (q782) qIs48] (I, III)*	CGC
ROG120	*htp-3-wrmScarlet (slc1) syp-3 I; ieSi11[syp-3p::EmeraldGFP::syp-3::syp-3 3'UTR + Cbr-unc-119(+)] II*	Cross between *htp-3-wrmScarlet* and *syp-3 I; ieSi11 II*
ROG212	*meIs8 [pie-1p::GFP::cosa-1 + unc-119(+)] II; pSUN-1::TIR-1::mRuby him-8(tm611) spo-11-aid (slc3) IV*	[41]
ROG198	*syp-1^{K42E} (slc11) V*	Created by CRISPR/Cas9 microinjections
ROG199	*syp-1$^{\Delta8}$ (slc12) V*	Created by CRISPR/Cas9 microinjections
ROG200	*htp-3 (tm3656) I; syp-1^{K42E} (slc11) V*	Cross between TY5038 and ROG198
ROG201	*htp-3 (tm3656) I; syp-1$^{\Delta8}$ (slc12) V*	Cross between TY5038 and ROG199
AV620	*meIs8 [pie-1p::GFP::cosa-1 + unc-119(+)] II*	CGC
ROG202	*meIs8 [pie-1p::GFP::cosa-1 + unc-119(+)] II; syp-1^{K42E} (slc11) V*	Cross between AV620 and ROG198
ROG210	*meIs8 [pie-1p::GFP::cosa-1 + unc-119(+)] II; syp-1$^{\Delta8}$ (slc12) V*	Cross between AV620 and ROG199
ROG208	*him-8(tm611) IV; syp-1$^{\Delta8}$ (slc12) V*	Cross between CA257 and ROG199
ROG211	*htp-3-wrmScarlet (slc1) syp-3 (ok758) I; ieSi11 [syp-3p::EmeraldGFP::syp-3::syp-3 3'UTR + Cbr-unc-119(+)] II; syp-1^{K42E} (slc11) V; nT1 [qIs51] (IV;V)*	Cross between ROG120 and ROG198
ROG250	*htp-3-wrmScarlet (slc1) syp-3 (ok758) I; ieSi11 [syp-3p::EmeraldGFP::syp-3::syp-3 3'UTR + Cbr-unc-119(+)] II; syp-1$^{\Delta8}$ (slc12) V; nT1 [qIs51] (IV;V)*	Cross between ROG120 and ROG199
ROG255	*syp-1$^{\Delta8}$ (slc12) V (Hawaiian)*	Microinjections into CB4856
ROG256	*syp-1^{K42E} (slc11) V (Hawaiian)*	Microinjections into CB4856
